# Supplementary material for: Mapping the HPV Landscape in South African Women: A Systematic Review and Meta-Analysis of Viral Genotypes, Microbiota, and Immune Signals
Source: Viruses. 2024 Dec 8;16(12):1893. doi: 10.3390/v16121893 (PMC11680443; doi:10.3390/v16121893)
Supplement: Supplementary file 1 [file viruses-16-01893-s001.zip › Figure S1_Prevalence by province-output.pdf]

Study	Events	Total	Proportion	95%-CI	Weight
**Province = Eastern Cape**					
taku et al. 2020	234	737	0.32	[0.28; 0.35]	0.9%
taku et al. 2020	119	417	0.29	[0.24; 0.33]	0.9%
mbulawa et al. 2021	162	213	0.76	[0.70; 0.82]	0.9%
taku et al. 2021	66	205	0.32	[0.26; 0.39]	0.9%
taku et al. 2021	185	193	0.96	[0.92; 0.98]	0.9%
onywera et al. 2022	162	213	0.76	[0.70; 0.82]	0.9%
mbulawa et al. 2022	383	455	0.84	[0.80; 0.87]	0.9%
**Random effects model**		**2433**	**0.63**	**[0.39; 0.84]**	**6.4%**
Heterogeneity: $I^2 = 99.3\%$, $\tau^2 = 0.1022$, $p < 0.0001$					
**Province = Gauteng**					
wright et al. 1995	8	9	0.89	[0.52; 1.00]	0.7%
boy et al. 2006	2	18	0.11	[0.01; 0.35]	0.8%
richter et al. 2008	35	60	0.58	[0.45; 0.71]	0.9%
firnhaber et al. 2009	140	148	0.95	[0.90; 0.98]	0.9%
said et al. 2009	89	153	0.58	[0.50; 0.66]	0.9%
firnhaber et al. 2010	191	1010	0.19	[0.17; 0.21]	0.9%
ngou et al. 2013	50	149	0.34	[0.26; 0.42]	0.9%
richter et al. 2013	1078	1445	0.75	[0.72; 0.77]	0.9%
vogt et al. 2013	35	68	0.51	[0.39; 0.64]	0.9%
adamson et al. 2015	116	316	0.37	[0.31; 0.42]	0.9%
lebelo et al. 2015	80	86	0.93	[0.85; 0.97]	0.9%
ngou et al. 2015	548	620	0.88	[0.86; 0.91]	0.9%
van aardt et al. 2015	274	299	0.92	[0.88; 0.95]	0.9%
firnhaber et al. 2016	225	688	0.33	[0.29; 0.36]	0.9%
mbulawa et al. 2016	720	1161	0.62	[0.59; 0.65]	0.9%
papasavvas et al. 2016	39	55	0.71	[0.57; 0.82]	0.9%
segondy et al. 2016	482	560	0.86	[0.83; 0.89]	0.9%
van aardt et al. 2016	251	270	0.93	[0.89; 0.96]	0.9%
goeieman et al. 2017	166	398	0.42	[0.37; 0.47]	0.9%
kelly et al. 2017	551	621	0.89	[0.86; 0.91]	0.9%
kelly et al. 2017	491	621	0.79	[0.76; 0.82]	0.9%
mbulawa et al. 2017	82	196	0.42	[0.35; 0.49]	0.9%
rad et al. 2017	159	167	0.95	[0.91; 0.98]	0.9%
chikandiwa et al. 2018	254	621	0.41	[0.37; 0.45]	0.9%
kelly et al. 2018	472	604	0.78	[0.75; 0.81]	0.9%
kremer et al. 2018	258	463	0.56	[0.51; 0.60]	0.9%
mbulawa et al. 2018	93	143	0.65	[0.57; 0.73]	0.9%
papasavvas et al. 2019	39	55	0.71	[0.57; 0.82]	0.9%
van de wijgert et al. 2020	497	623	0.80	[0.76; 0.83]	0.9%
wood et al. 2020	2	116	0.02	[0.00; 0.06]	0.9%
kelly et al. 2021	456	575	0.79	[0.76; 0.83]	0.9%
tiiti et al. 2021	253	527	0.48	[0.44; 0.52]	0.9%
tiiti et al. 2021	253	527	0.48	[0.44; 0.52]	0.9%
tiiti et al. 2022	253	526	0.48	[0.44; 0.52]	0.9%
maroga et al. 2023	78	101	0.77	[0.68; 0.85]	0.9%
snyman et al 2023	282	903	0.31	[0.28; 0.34]	0.9%
vink et al. 2023	199	411	0.48	[0.43; 0.53]	0.9%
maueia et al. 2024	52	52	1.00	[0.93; 1.00]	0.9%
**Random effects model**		**15365**	**0.64**	**[0.55; 0.73]**	**34.0%**
Heterogeneity: $I^2 = 99.1\%$, $\tau^2 = 0.0868$, $p = 0$					
**Province = KwaZulu-Natal**					
cooper et al. 1991	44	69	0.64	[0.51; 0.75]	0.9%
cooper et al. 1991	36	72	0.50	[0.38; 0.62]	0.9%
cooper et al. 1992	9	22	0.41	[0.21; 0.64]	0.8%
denny et al. 2008	301	397	0.76	[0.71; 0.80]	0.9%
moodley et al. 2010	75	124	0.60	[0.51; 0.69]	0.9%
auvert et al. 2011	62	88	0.70	[0.60; 0.80]	0.9%
de vuyst et al. 2012	96	109	0.88	[0.80; 0.93]	0.9%
ebrahim et al. 2016	171	224	0.76	[0.70; 0.82]	0.9%
mbatha et al. 2017	301	1223	0.25	[0.22; 0.27]	0.9%
mbatha et al. 2017	37	98	0.38	[0.28; 0.48]	0.9%
liebenberg et al. 2019	575	779	0.74	[0.71; 0.77]	0.9%
naidoo et al 2022	108	190	0.57	[0.49; 0.64]	0.9%
qulu et al. 2023	83	243	0.34	[0.28; 0.40]	0.9%
downham et al 2024	30	241	0.12	[0.09; 0.17]	0.9%
sebitloane et al. 2024	98	350	0.28	[0.23; 0.33]	0.9%
**Random effects model**		**4229**	**0.53**	**[0.41; 0.65]**	**13.4%**
Heterogeneity: $I^2 = 98.7\%$, $\tau^2 = 0.0571$, $p < 0.0001$					
**Province = Limpopo**					
dols et al. 2012	30	51	0.59	[0.44; 0.72]	0.9%
**Province = Western Cape**					
williamson et al. 1989	65	98	0.66	[0.56; 0.76]	0.9%
padayachee et al. 1993	12	12	1.00	[0.74; 1.00]	0.8%
williamson et al. 1994	55	68	0.81	[0.70; 0.89]	0.9%
ramesar et al. 1996	29	206	0.14	[0.10; 0.20]	0.9%
kuhn et al. 2000	477	2943	0.16	[0.15; 0.18]	0.9%
wright et al. 2000	302	1415	0.21	[0.19; 0.24]	0.9%
denny et al. 2000	214	1335	0.16	[0.14; 0.18]	0.9%
kay et al. 2002	23	31	0.74	[0.55; 0.88]	0.8%
passmore et al. 2002	5	8	0.62	[0.24; 0.91]	0.7%
kay et al. 2003	161	179	0.90	[0.85; 0.94]	0.9%
kay et al. 2005	89	554	0.16	[0.13; 0.19]	0.9%
allan et al. 2006	254	1491	0.17	[0.15; 0.19]	0.9%
passmore et al. 2006	30	33	0.91	[0.76; 0.98]	0.8%
jones et al. 2007	163	450	0.36	[0.32; 0.41]	0.9%
passmore et al. 2007	114	206	0.55	[0.48; 0.62]	0.9%
allan et al. 2008	305	1073	0.28	[0.26; 0.31]	0.9%
marais et al. 2008	122	214	0.57	[0.50; 0.64]	0.9%
marais et al. 2008	261	1003	0.26	[0.23; 0.29]	0.9%
mbulawa et al. 2009	132	252	0.52	[0.46; 0.59]	0.9%
moodley et al. 2009	97	109	0.89	[0.82; 0.94]	0.9%
denny et al. 2010	1394	6637	0.21	[0.20; 0.22]	0.9%
kuhn et al. 2010	1402	6552	0.21	[0.20; 0.22]	0.9%
mbulawa et al. 2010	281	484	0.58	[0.54; 0.62]	0.9%
taylor et al. 2010	75	303	0.25	[0.20; 0.30]	0.9%
taylor et al. 2011	355	1858	0.19	[0.17; 0.21]	0.9%
wang et al. 2011	933	5595	0.17	[0.16; 0.18]	0.9%
mbulawa et al. 2012	281	484	0.58	[0.54; 0.62]	0.9%
mcdonald et al. 2012	1675	8050	0.21	[0.20; 0.22]	0.9%
meiring et al. 2012	99	109	0.91	[0.84; 0.96]	0.9%
adler et al. 2014	55	85	0.65	[0.54; 0.75]	0.9%
ameur et al. 2014	20	20	1.00	[0.83; 1.00]	0.8%
bere et al. 2014	67	67	1.00	[0.95; 1.00]	0.9%
mbulawa et al. 2014	136	440	0.31	[0.27; 0.35]	0.9%
mcdonald et al. 2014	2389	9421	0.25	[0.24; 0.26]	0.9%
adler et al. 2015	43	83	0.52	[0.41; 0.63]	0.9%
mbulawa et al. 2015	281	484	0.58	[0.54; 0.62]	0.9%
tayib et al. 2015	106	113	0.94	[0.88; 0.97]	0.9%
zeier et al. 2015	287	300	0.96	[0.93; 0.98]	0.9%
adler et al. 2016	64	100	0.64	[0.54; 0.73]	0.9%
kriek et al. 2016	83	165	0.50	[0.42; 0.58]	0.9%
mbatani et al. 2016	47	50	0.94	[0.83; 0.99]	0.9%
mbulawa et al. 2018	101	148	0.68	[0.60; 0.76]	0.9%
onywera et al. 2019	37	87	0.43	[0.32; 0.54]	0.9%
chambuso et al. 2020	148	173	0.86	[0.79; 0.90]	0.9%
johnson et al. 2020	222	714	0.31	[0.28; 0.35]	0.9%
kuhn et al. 2020	556	715	0.78	[0.75; 0.81]	0.9%
onywera et al. 2021	37	87	0.43	[0.32; 0.54]	0.9%
saidu et al. 2021	220	705	0.31	[0.28; 0.35]	0.9%
denny et al. 2023	857	3062	0.28	[0.26; 0.30]	0.9%
happel et al. 2023	20	33	0.61	[0.42; 0.77]	0.8%
habour et al. 2024	2	12	0.17	[0.02; 0.48]	0.8%
**Random effects model**		**58816**	**0.53**	**[0.44; 0.62]**	**45.3%**
Heterogeneity: $I^2 = 99.2\%$, $\tau^2 = 0.1050$, $p = 0$					
**Random effects model**		**80894**	**0.57**	**[0.52; 0.63]**	**100.0%**
**Prediction interval**				**[0.06; 0.99]**	
Heterogeneity: $I^2 = 99.4\%$, $\tau^2 = 0.0917$, $p = 0$					
Test for subgroup differences: $\chi^2_4 = 3.48$, df = 4 ($p = 0.4810$)					

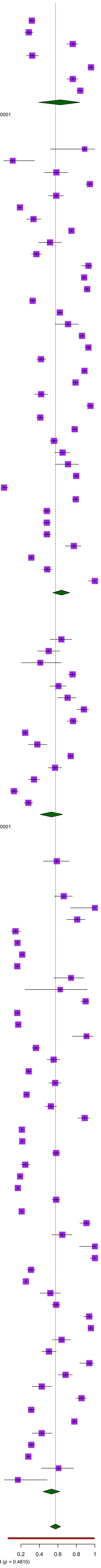

Figure S1: Forest plot showing the prevalence of HPV among women in various provinces of South Africa based on different studies. The random effects model estimated an overall prevalence with significant heterogeneity ($I^2 = 99\%$, $^2 = 0.0917$, $p < 0.001$). Subgroup analysis did not show significant differences in HPV prevalence between provinces ($^2 = 3.48$, df = 4, $p = 0.48$).
