# Supplementary material for: Mapping the HPV Landscape in South African Women: A Systematic Review and Meta-Analysis of Viral Genotypes, Microbiota, and Immune Signals
Source: Viruses. 2024 Dec 8;16(12):1893. doi: 10.3390/v16121893 (PMC11680443; doi:10.3390/v16121893)
Supplement: Supplementary file 1 [file viruses-16-01893-s001.zip › Figure S2_Prevalence by sample type-output.pdf]

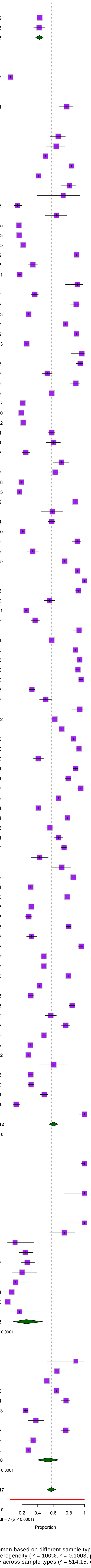

Study	Events	Total	Proportion	95%-CI	Weight
**sample type = Anal**					
goeieman et al. 2017	85	199	0.43	[0.36; 0.50]	0.8%
mbulawa et al. 2017	82	196	0.42	[0.35; 0.49]	0.8%
**Random effects model**		**395**	**0.42**	**[0.37; 0.47]**	**1.6%**
Heterogeneity: $I^2 = 0\%$, $\tau^2 = 0$, $p = 0.8606$					
**sample type = Blood**					
kay et al. 2005	12	227	0.05	[0.03; 0.09]	0.8%
**sample type = Breast tissue**					
maroga et al. 2023	78	101	0.77	[0.68; 0.85]	0.8%
**sample type = Cervical**					
williamson et al. 1989	65	98	0.66	[0.56; 0.76]	0.8%
cooper et al. 1991	44	69	0.64	[0.51; 0.75]	0.8%
cooper et al. 1991	36	72	0.50	[0.38; 0.62]	0.8%
johnson et al. 1991	10	12	0.83	[0.52; 0.98]	0.7%
cooper et al. 1992	9	22	0.41	[0.21; 0.64]	0.7%
williamson et al. 1994	55	68	0.81	[0.70; 0.89]	0.8%
grayson et al. 1996	8	11	0.73	[0.39; 0.94]	0.7%
ramesar et al. 1996	29	206	0.14	[0.10; 0.20]	0.8%
chetty et al. 1997	32	50	0.64	[0.49; 0.77]	0.8%
denny et al. 2000	214	1335	0.16	[0.14; 0.18]	0.8%
kuhn et al. 2000	477	2943	0.16	[0.15; 0.18]	0.8%
wright et al. 2000	302	1415	0.21	[0.19; 0.24]	0.8%
kay et al. 2003	161	179	0.90	[0.85; 0.94]	0.8%
kay et al. 2005	77	227	0.34	[0.28; 0.40]	0.8%
allan et al. 2006	254	1491	0.17	[0.15; 0.19]	0.8%
passmore et al. 2006	30	33	0.91	[0.76; 0.98]	0.8%
jones et al. 2007	163	450	0.36	[0.32; 0.41]	0.8%
passmore et al. 2007	92	103	0.89	[0.82; 0.95]	0.8%
allan et al. 2008	305	1073	0.28	[0.26; 0.31]	0.8%
denny et al. 2008	301	397	0.76	[0.71; 0.80]	0.8%
marais et al. 2008	98	109	0.90	[0.83; 0.95]	0.8%
marais et al. 2008	261	1003	0.26	[0.23; 0.29]	0.8%
richter et al. 2008	29	30	0.97	[0.83; 1.00]	0.8%
firnhaber et al. 2009	140	148	0.95	[0.90; 0.98]	0.8%
mbulawa et al. 2009	132	252	0.52	[0.46; 0.59]	0.8%
moodley et al. 2009	97	109	0.89	[0.82; 0.94]	0.8%
said et al. 2009	89	153	0.58	[0.50; 0.66]	0.8%
denny et al. 2010	1394	6637	0.21	[0.20; 0.22]	0.8%
firnhaber et al. 2010	191	1010	0.19	[0.17; 0.21]	0.8%
kuhn et al. 2010	1402	6552	0.21	[0.20; 0.22]	0.8%
mbulawa et al. 2010	281	484	0.58	[0.54; 0.62]	0.8%
moodley et al. 2010	75	124	0.60	[0.51; 0.69]	0.8%
taylor et al. 2010	75	303	0.25	[0.20; 0.30]	0.8%
auvert et al. 2011	62	88	0.70	[0.60; 0.80]	0.8%
firnhaber et al. 2011	98	157	0.62	[0.54; 0.70]	0.8%
taylor et al. 2011	355	1858	0.19	[0.17; 0.21]	0.8%
wang et al. 2011	933	5595	0.17	[0.16; 0.18]	0.8%
de vuyst et al. 2012	96	109	0.88	[0.80; 0.93]	0.8%
dols et al. 2012	30	51	0.59	[0.44; 0.72]	0.8%
mbulawa et al. 2012	281	484	0.58	[0.54; 0.62]	0.8%
mcdonald et al. 2012	1675	8050	0.21	[0.20; 0.22]	0.8%
meiring et al. 2012	99	109	0.91	[0.84; 0.96]	0.8%
ngou et al. 2013	50	149	0.34	[0.26; 0.42]	0.8%
richter et al. 2013	1078	1445	0.75	[0.72; 0.77]	0.8%
vogt et al. 2013	31	34	0.91	[0.76; 0.98]	0.8%
ameur et al. 2014	20	20	1.00	[0.83; 1.00]	0.7%
denny et al. 2014	210	228	0.92	[0.88; 0.95]	0.8%
mbulawa et al. 2014	121	219	0.55	[0.48; 0.62]	0.8%
mcdonald et al. 2014	2389	9421	0.25	[0.24; 0.26]	0.8%
adamson et al. 2015	116	316	0.37	[0.31; 0.42]	0.8%
lebelo et al. 2015	80	86	0.93	[0.85; 0.97]	0.8%
mbulawa et al. 2015	281	484	0.58	[0.54; 0.62]	0.8%
ngou et al. 2015	548	620	0.88	[0.86; 0.91]	0.8%
tayib et al. 2015	106	113	0.94	[0.88; 0.97]	0.8%
van aardt et al. 2015	274	299	0.92	[0.88; 0.95]	0.8%
zeier et al. 2015	287	300	0.96	[0.93; 0.98]	0.8%
firnhaber et al. 2016	225	688	0.33	[0.29; 0.36]	0.8%
kriek et al. 2016	83	165	0.50	[0.42; 0.58]	0.8%
mbatani et al. 2016	47	50	0.94	[0.83; 0.99]	0.8%
mbulawa et al. 2016	1440	2322	0.62	[0.60; 0.64]	0.8%
papasavvas et al. 2016	39	55	0.71	[0.57; 0.82]	0.8%
segondy et al. 2016	482	560	0.86	[0.83; 0.89]	0.8%
van aardt et al. 2016	251	270	0.93	[0.89; 0.96]	0.8%
goeieman et al. 2017	81	199	0.41	[0.34; 0.48]	0.8%
kelly et al. 2017	551	621	0.89	[0.86; 0.91]	0.8%
kelly et al. 2017	491	621	0.79	[0.76; 0.82]	0.8%
rad et al. 2017	159	167	0.95	[0.91; 0.98]	0.8%
barnabas et al. 2018	199	298	0.67	[0.61; 0.72]	0.8%
chikandiwa et al. 2018	254	621	0.41	[0.37; 0.45]	0.8%
kelly et al. 2018	472	604	0.78	[0.75; 0.81]	0.8%
kremer et al. 2018	258	463	0.56	[0.51; 0.60]	0.8%
mbulawa et al. 2018	194	291	0.67	[0.61; 0.72]	0.8%
liebenberg et al. 2019	575	779	0.74	[0.71; 0.77]	0.8%
onywera et al. 2019	37	87	0.43	[0.32; 0.54]	0.8%
papasavvas et al. 2019	39	55	0.71	[0.57; 0.82]	0.8%
chambuso et al. 2020	148	173	0.86	[0.79; 0.90]	0.8%
johnson et al. 2020	222	714	0.31	[0.28; 0.35]	0.8%
kuhn et al. 2020	556	715	0.78	[0.75; 0.81]	0.8%
taku et al. 2020	234	737	0.32	[0.28; 0.35]	0.8%
taku et al. 2020	119	417	0.29	[0.24; 0.33]	0.8%
van de wijgert et al. 2020	497	623	0.80	[0.76; 0.83]	0.8%
taku et al. 2021	66	205	0.32	[0.26; 0.39]	0.8%
taku et al. 2021	185	193	0.96	[0.92; 0.98]	0.8%
tiiti et al. 2021	253	527	0.48	[0.44; 0.52]	0.8%
tiiti et al. 2021	253	527	0.48	[0.44; 0.52]	0.8%
kelly et al. 2021	456	575	0.79	[0.76; 0.83]	0.8%
onywera et al. 2021	37	87	0.43	[0.32; 0.54]	0.8%
saidu et al. 2021	220	705	0.31	[0.28; 0.35]	0.8%
mbulawa et al. 2022	383	455	0.84	[0.80; 0.87]	0.8%
naidoo et al 2022	108	190	0.57	[0.49; 0.64]	0.8%
onywera et al. 2022	162	213	0.76	[0.70; 0.82]	0.8%
tiiti et al. 2022	253	526	0.48	[0.44; 0.52]	0.8%
botha et al. 2023	275	899	0.31	[0.28; 0.34]	0.8%
denny et al. 2023	857	3062	0.28	[0.26; 0.30]	0.8%
happel et al. 2023	20	33	0.61	[0.42; 0.77]	0.8%
snyman et al 2023	282	903	0.31	[0.28; 0.34]	0.8%
sørbye et al. 2023	224	710	0.32	[0.28; 0.35]	0.8%
vink et al. 2023	199	411	0.48	[0.43; 0.53]	0.8%
downham et al 2024	30	241	0.12	[0.09; 0.17]	0.8%
maueia et al. 2024	52	52	1.00	[0.93; 1.00]	0.8%
**Random effects model**		**80212**	**0.60**	**[0.54; 0.66]**	**80.0%**
Heterogeneity: $I^2 = 99.5\%$, $\tau^2 = 0.0898$, $p = 0$					
**sample type = Genital wart biopsy**					
bere et al. 2014	77	77	1.00	[0.95; 1.00]	0.8%
**sample type = Laryngeal**					
padayachee et al. 1993	12	12	1.00	[0.74; 1.00]	0.7%
**sample type = Oral**					
padayachee et al. 1991	7	7	1.00	[0.59; 1.00]	0.6%
kay et al. 2002	23	31	0.74	[0.55; 0.88]	0.8%
boy et al. 2006	2	18	0.11	[0.01; 0.35]	0.7%
passmore et al. 2007	22	91	0.24	[0.16; 0.34]	0.8%
marais et al. 2008	28	105	0.27	[0.19; 0.36]	0.8%
richter et al. 2008	6	30	0.20	[0.08; 0.39]	0.8%
vogt et al. 2013	4	34	0.12	[0.03; 0.27]	0.8%
mbulawa et al. 2014	15	221	0.07	[0.04; 0.11]	0.8%
wood et al. 2020	2	116	0.02	[0.00; 0.06]	0.8%
habour et al. 2024	2	12	0.17	[0.02; 0.48]	0.7%
**Random effects model**		**665**	**0.25**	**[0.09; 0.47]**	**7.5%**
Heterogeneity: $I^2 = 93.6\%$, $\tau^2 = 0.1103$, $p < 0.0001$					
**sample type = Vaginal**					
wright et al. 1995	8	9	0.89	[0.52; 1.00]	0.6%
adler et al. 2014	55	85	0.65	[0.54; 0.75]	0.8%
adler et al. 2015	43	83	0.52	[0.41; 0.63]	0.8%
adler et al. 2016	64	100	0.64	[0.54; 0.73]	0.8%
ebrahim et al. 2016	171	224	0.76	[0.70; 0.82]	0.8%
mbatha et al. 2017	301	1223	0.25	[0.22; 0.27]	0.8%
mbatha et al. 2017	37	98	0.38	[0.28; 0.48]	0.8%
mbulawa et al. 2021	162	213	0.76	[0.70; 0.82]	0.8%
qulu et al. 2023	83	243	0.34	[0.28; 0.40]	0.8%
sebitloane et al. 2024	98	350	0.28	[0.23; 0.33]	0.8%
**Random effects model**		**2628**	**0.53**	**[0.39; 0.67]**	**7.9%**
Heterogeneity: $I^2 = 98.1\%$, $\tau^2 = 0.0475$, $p < 0.0001$					
**Random effects model**		**84317**	**0.57**	**[0.52; 0.63]**	**100.0%**
**Prediction interval**				**[0.05; 1.00]**	
Heterogeneity: $I^2 = 99.4\%$, $\tau^2 = 0.1003$, $p = 0$					
Test for subgroup differences: $\chi^2_7 = 514.15$, df = 7 ($p < 0.0001$)					

Figure S2: Prevalence of HPV among women based on different sample types. The random-effects model estimated an overall prevalence with significant heterogeneity (I^2 = 100%, ^{2} = 0.1003, p < 0.001). Subgroup analysis revealed significant differences in HPV prevalence across sample types (^{2} = 514.15, df = 7, p < 0.01).
