# Supplementary material for: Mapping the HPV Landscape in South African Women: A Systematic Review and Meta-Analysis of Viral Genotypes, Microbiota, and Immune Signals
Source: Viruses. 2024 Dec 8;16(12):1893. doi: 10.3390/v16121893 (PMC11680443; doi:10.3390/v16121893)
Supplement: Supplementary file 1 [file viruses-16-01893-s001.zip › Figure S3_Prevalence by HIV status-output_.pdf]

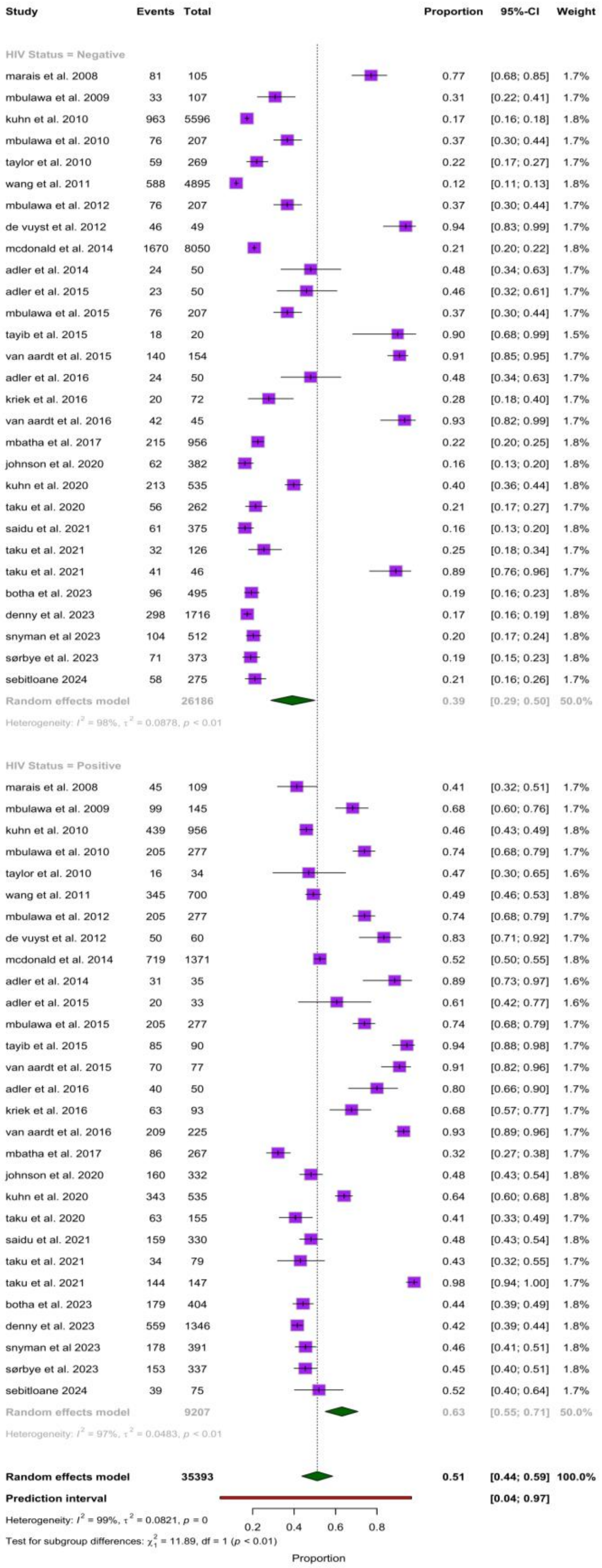

Figure S3: Forest plot indicating the prevalence of HPV among South African women based on HIV status. The estimated prevalence from the random effects model showed a significant heterogeneity (I² = 99%, τ² = 0.0821, p < 0.001).
