# Supplementary material for: Mapping the HPV Landscape in South African Women: A Systematic Review and Meta-Analysis of Viral Genotypes, Microbiota, and Immune Signals
Source: Viruses. 2024 Dec 8;16(12):1893. doi: 10.3390/v16121893 (PMC11680443; doi:10.3390/v16121893)
Supplement: Supplementary file 1 [file viruses-16-01893-s001.zip › Figure S4_.pdf]

A

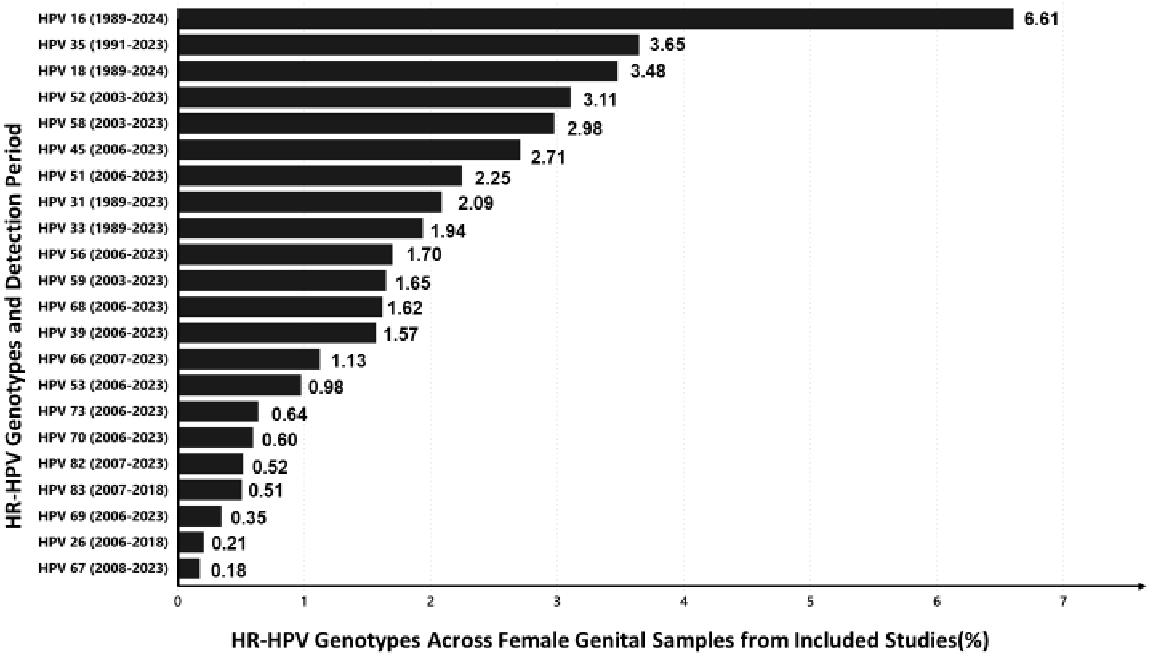

B

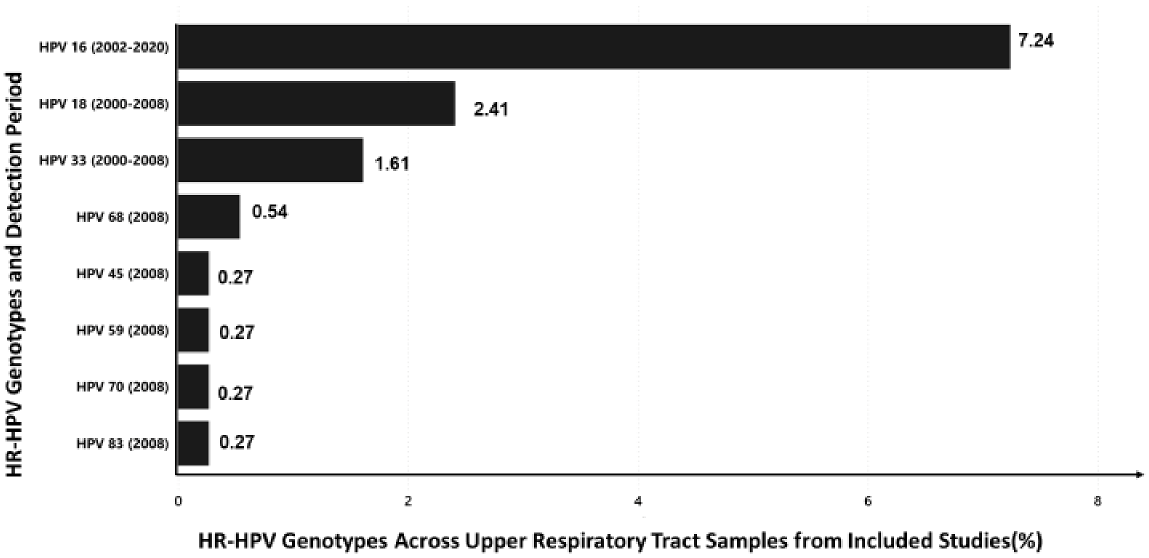

Figure S4: Prevalence (%) of HR-HPV genotypes detected in the FGT (A) and URT (B) samples from 1989 to 2024, and 2002 to 2020, respectively.
