# Supplementary material for: Mapping the HPV Landscape in South African Women: A Systematic Review and Meta-Analysis of Viral Genotypes, Microbiota, and Immune Signals
Source: Viruses. 2024 Dec 8;16(12):1893. doi: 10.3390/v16121893 (PMC11680443; doi:10.3390/v16121893)
Supplement: Supplementary file 1 [file viruses-16-01893-s001.zip › Figure S5_.pdf]

A

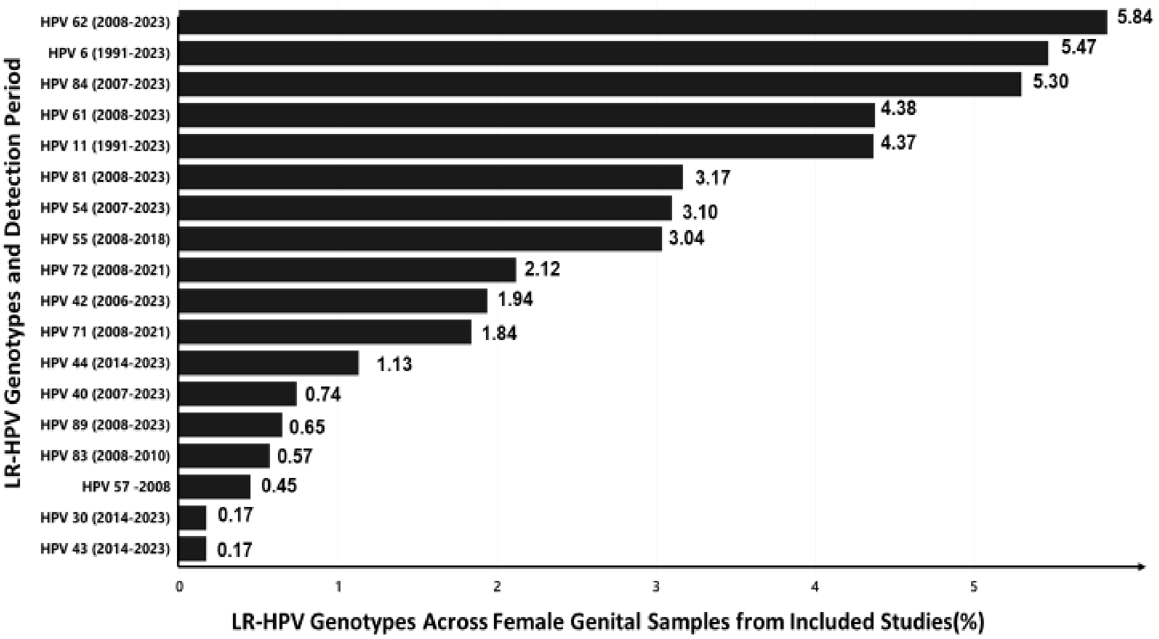

B

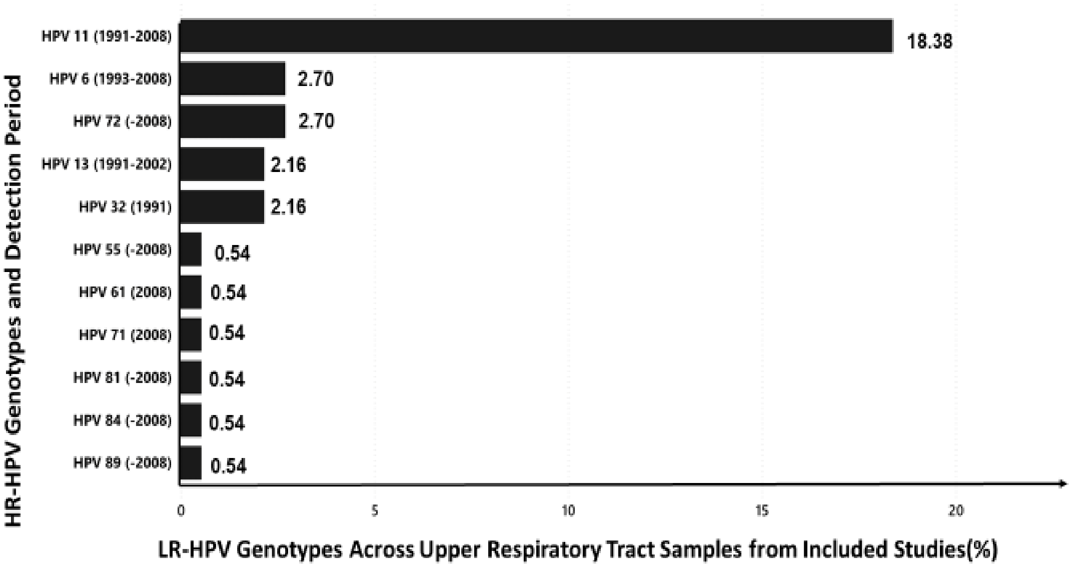

Figure S5: Prevalence (%) of LR-HPV genotypes detected in the FGT (A) and URT (B) samples detected from 1991 to 2023, and 1991 to 2008, respectively.
