# Supplementary material for: Mapping the HPV Landscape in South African Women: A Systematic Review and Meta-Analysis of Viral Genotypes, Microbiota, and Immune Signals
Source: Viruses. 2024 Dec 8;16(12):1893. doi: 10.3390/v16121893 (PMC11680443; doi:10.3390/v16121893)
Supplement: Supplementary file 1 [file viruses-16-01893-s001.zip › Figure S6.pdf]

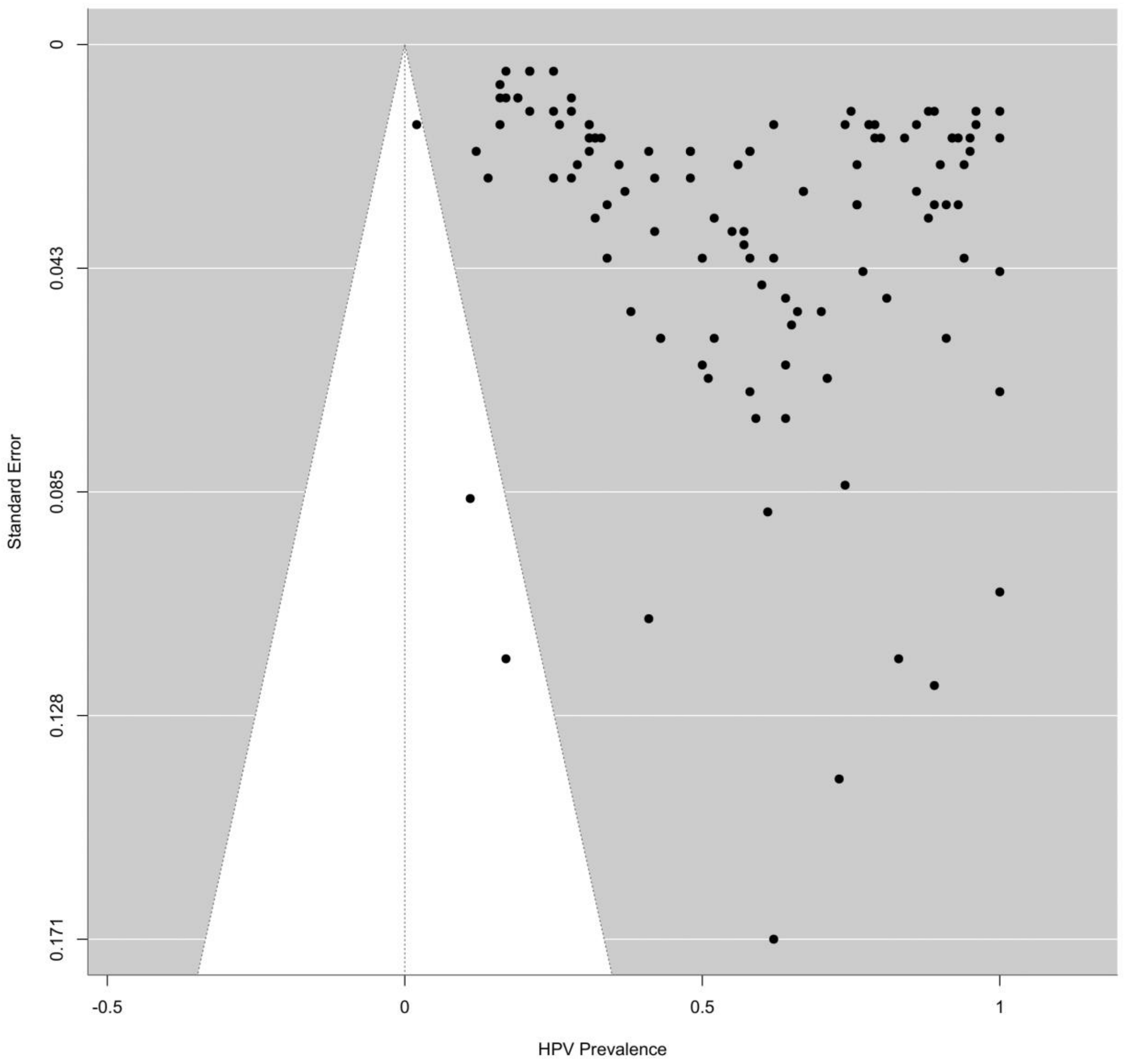

Figure S6: The funnel plot displays the relationship between HPV prevalence (x-axis) and the standard error (y-axis) across the included studies assessing publication bias in HPV prevalence studies.
