# Supplementary material for: Mapping the HPV Landscape in South African Women: A Systematic Review and Meta-Analysis of Viral Genotypes, Microbiota, and Immune Signals
Source: Viruses. 2024 Dec 8;16(12):1893. doi: 10.3390/v16121893 (PMC11680443; doi:10.3390/v16121893)
Supplement: Supplementary file 1 [file viruses-16-01893-s001.zip › JBL Score tables.pdf]

Table S2: Cross-Sec onal

Study ID	Q1	Q2	Q3	Q4	Q5	Q6	Q7	Q8	Total	Overall Judgment
**Adamson et al. 2015**	Y	Y	Y	Y	U	U	Y	Y	6	Moderate risk
**Adler et al. 2014**	Y	Y	Y	Y	Y	U	Y	Y	7	Low risk
**Ameur et al. 2014**	Y	Y	Y	Y	Y	U	Y	U	6	Moderate risk
**Bere et al. 2014**	Y	Y	Y	Y	Y	Y	Y	Y	8	Low risk
**Botha et al. 2023**	Y	Y	Y	Y	Y	Y	Y	Y	8	Low risk
**Boy et al. 2006**	Y	Y	Y	Y	N	U	Y	Y	6	Moderate risk
**Chambuso et al. 2020**	Y	Y	Y	Y	Y	Y	Y	Y	8	Low risk
**Chetty et al. 1997**	Y	Y	Y	Y	Y	N	Y	Y	7	Low risk
**Cooper et al. 1991**	Y	Y	Y	Y	U	U	Y	Y	6	Moderate risk
**Denny et al. 2000**	Y	Y	Y	Y	Y	U	Y	Y	7	Low risk
**Denny et al. 2014**	Y	Y	Y	Y	Y	U	Y	Y	7	Low risk
**Denny et al. 2023**	Y	Y	Y	Y	U	U	Y	Y	6	Moderate risk
**Dols et al. 2012**	Y	Y	Y	Y	U	U	Y	Y	6	Moderate risk
**Downham et al. 2024**	Y	Y	Y	Y	U	U	Y	Y	6	Moderate risk

**Firnhaber et al. 2009**	Y	Y	Y	Y	Y	U	Y	Y	6	Moderate risk
**Firnhaber et al. 2010**	Y	Y	Y	Y	Y	Y	Y	Y	8	Low risk
**Firnhaber et al. 2011**	Y	Y	Y	Y	Y	Y	Y	Y	8	Low risk
**Goeieman et al. 2017**	Y	Y		Y	Y	Y	Y	Y	8	Low risk
**Jones et al. 2007**	Y	Y	Y	Y	U	U	Y	Y	6	Moderate risk
**Kay et al. 2002**	Y	Y	Y	Y	U	U	Y	U	5	Moderate risk
**Kay et al. 2003**	Y	Y	Y	Y	U	N	Y	Y	6	Moderate risk
**Kremer et al. 2018**	Y	Y	Y	Y	Y	Y	Y	Y	8	Low risk
**Kuhn et al. 2000**	Y	Y	Y	Y	Y	U	Y	Y	7	Low risk
**Lebelo et al. 2015**	Y	Y	Y	Y	Y	U	Y	Y	7	Low risk
**Marais et al. 2008**	Y	Y	Y	Y	U	U	Y	Y	6	Moderate risk
**Maroga et al. 2023**	Y	Y	Y	Y	U	U	Y	Y	6	Moderate risk
**Maueia et al. 2024**	Y	Y	Y	Y	Y	U	Y	Y	7	Low risk
**Mbatha et al.2017**	Y	Y	Y	Y	N	N	Y	Y	6	Moderate risk
**Mbatha et al.2017**	Y	Y	Y	Y	Y	Y	Y	Y	8	Low risk
**Mbulawa et al.2009**	Y	Y	Y	Y	Y	Y	Y	Y	8	Low risk

**Mbulawa et al.2010**	Y	Y	Y	Y	Y	U	Y	Y	7	Low risk
**Mbulawa et al.2015**	Y	Y	Y	Y	N	N	Y	Y	6	Moderate risk
**Mbulawa et al.2022**	Y	Y	Y	Y	Y	N	Y	Y	7	Low risk
**Mbulawa et al.2014**	Y	U	Y	Y	Y	Y	Y	Y	7	Low risk
**Mbulawa et al.2018**	Y	Y	Y	Y	Y	Y	Y	Y	8	Low risk
**Mbulawa et al.2021**	Y	Y	Y	Y	Y	U	Y	Y	7	Low risk
**Meiring et al.2012**	Y	Y	Y	Y	U	N	Y	N	5	Moderate risk
**Moodle et al.2009**	Y	Y	Y	Y	Y	Y	Y	Y	8	Low risk
**Moodle et al.2010**	Y	Y	Y	Y	Y	Y	Y	Y	8	Low risk
**Moroga et al.2023**	Y	Y	Y	Y	N	N	Y	Y	6	Moderate risk
**Ngou et al.2013**	Y	Y	Y	Y	U	U	Y	Y	6	Moderate risk
**Ngou et al.2015**	Y	Y	Y	Y	U	U	Y	Y	6	Moderate risk
**Onywera et al.2019**	Y	Y	Y	Y	Y	U	Y	Y	7	Low risk
**Onywera et al. 2022**	Y	Y	Y	Y	Y	N	Y	Y	7	Low risk
**Padayachee et al. 1991**	N	N/A	Y	N	N	N	Y	N	2	High risk
**Papasavvas et al.2016**	Y	Y	Y	Y	Y	N	Y	Y	7	Low risk

**Papasavvas et al.2019**	Y	Y	Y	Y	Y	U	Y	Y	7	Low risk
**Passmore et al.2007**	Y	Y	Y	Y	U	N	Y	N	5	Moderate risk
**Qulu et al.2023**	Y	Y	Y	Y	Y	Y	Y	Y	8	Low risk
**Ramesar et al. 1996**	Y	Y	Y	Y	U	N	Y	N	5	Moderate risk
**Richter et al.2008**	Y	Y	Y	Y	U	N	Y	N	5	Moderate risk
**Richter et al. 2013**	Y	Y	Y	Y	U	N	Y	N	5	Moderate risk
**Said et al. 2009**	Y	Y	Y	Y	U	N	Y	Y	6	Moderate risk
**Taku et al. 2020**	Y	Y	Y	Y	Y	N	Y	Y	7	Low risk
**Taku et al.2020**	Y	Y	Y	Y	Y	Y	Y	Y	8	Low risk
**Taku et al.2021**	Y	Y	Y	Y	U	U	Y	Y	6	Moderate risk
**Taku et al.2021**	Y	Y	Y	Y	U	N	Y	Y	6	Moderate risk
**Tiiti et al. 2021**	Y	Y	Y	Y	U	N	Y	Y	6	Moderate risk
**Tiiti et al. 2022**	Y	Y	Y	Y	Y	Y	Y	Y	8	Low risk
**Williamson et al. 1989**	N	N/A	Y	Y	U	N	Y	N	3	High risk
**Williamson et al. 1994**	Y	Y	Y	Y	N	N	Y	U	5	Moderate risk
**Vogst et al. 2013**	Y	Y	Y	Y	Y	N	Y	Y	7	Low risk

**Wood et al. 2020**	Y	Y	Y	Y	Y	N	Y	Y	7	Low risk
**Wright et al. 2000**	Y	Y	Y	Y	U	N	Y	U	5	Moderate risk

Y: Yes N: No N/A: Not applicable U: Unclear

Q1. Were the criteria for inclusion in the sample clearly defined?

Q2. Were the study subjects and the setting described in detail?

Q3. Was the exposure measured in a valid and reliable way?

Q4. Were objective, standard criteria used for measurement of the condition?

Q5. Were confounding factors identified?

Q6. Were strategies to deal with confounding factors stated?

Q7. Were the outcomes measured in a valid and reliable way?

Q8. Was appropriate statistical analysis used?

Table S3: Cohort

**Study ID**	**Q1**	**Q2**	**Q3**	**Q4**	**Q5**	**Q6**	**Q7**	**Q8**	**Q9**	**Q10**	**Q11**	**Total**	**Overall Judgment**
**Adler et al. 2015**	Y	Y	Y	Y	U	Y	Y	Y	N	N	Y	7	Moderate risk
**Adler et al. 2016**	Y	Y	Y	Y	U	Y	Y	U	N	U	Y	7	Moderate risk
**Auvert et al. 2011**	Y	Y	Y	Y	Y	Y	Y	Y	N	U	Y	9	Moderate risk
**Barnabas et al. 2017**	N	Y	Y	Y	N	N	Y	Y	N	N	Y	6	Moderate risk
**Chikandiwa et al. 2018**	Y	Y	Y	Y	Y	Y	Y	Y	N	N	Y	9	Low risk

**Cooper et al. 1991**	N	Y	Y	U	U	Y	Y	N/A	N/A	N	U	4	High risk
**Denny et al. 2008**	Y	Y	Y	Y	Y	Y	Y	Y	Y	Y	Y	11	Low risk
**Denny et al. 2010**	Y	Y	Y	U	U	Y	Y	Y	Y	N	Y	10	Low risk
**Ebrahim et al. 2016**	Y	U	Y	Y	Y	Y	Y	U	U	N	Y	7	Moderate risk
**Firbhaber et al.2016**	Y	Y	Y	N	N	Y	Y	Y	N	N	Y	7	Moderate risk
**Happel et al. 2023**	Y	Y	Y	Y	Y	U	Y	Y	U	U	Y	8	Moderate risk
**Habour et al. 2024**	Y	Y	Y	Y	U	Y	Y	N/A	N/A	N/A	Y	7	Moderate risk
**Johnson et al.2020**	Y	Y	Y	Y	Y	Y	Y	Y	N	N	Y	9	Low risk
**Kelly et al. 2017**	Y	Y	Y	Y	U	Y	Y	Y	U	N	Y	8	Moderate risk
**Kelly et al. 2017**	Y	Y	Y	Y	Y	Y	Y	Y	U	U	Y	9	Low risk
**Kelly et al. 2018**	Y	Y	Y	Y	Y	Y	Y	Y	U	U	Y	9	Low risk
**Kelly et al. 2021**	Y	Y	Y	Y	Y	Y	Y	Y	U	U	Y	9	Low risk
**Kriek et al. 2016**	Y	Y	Y	Y	Y	Y	Y	Y	U	U	Y	9	Low risk
**Kuhn et al. 2010**	Y	Y	Y	Y	Y	Y	Y	Y	U	U	Y	9	Low risk
**Kuhn et al. 2020**	Y	Y	Y	Y	Y	Y	Y	U	U	N	Y	8	Moderate risk
**Liebenberg et al. 2019**	Y	Y	Y	Y	Y	Y	Y	Y	U	U	Y	9	Low risk
**Mbulawa et al.2012**	Y	Y	Y	Y	Y	Y	Y	Y	U	N	Y	9	Low risk

**Mbulawa et al.2016**	N	Y	Y	U	N	Y	Y	U	N	N	U	4	High risk
**Mbulawa et al.2017**	N	N	Y	Y	Y	y	Y	N	U	N	Y	6	Moderate risk
**McDonald et al.2012**	Y	Y	Y	Y	Y	Y	Y	Y	U	U	Y	9	Low risk
**McDonald et al.2014**	Y	Y	Y	Y	N	Y	Y	Y	N	N	Y	8	Moderate risk
**Naidoo et al. 2022**	Y	Y	Y	Y	Y	Y	Y	Y	U	N	Y	9	Low risk
**Onywera et al.2021**	Y	Y	Y	Y	Y	Y	N	N	N	N	U	6	Moderate risk
**Passmore et al.2002**	Y	Y	Y	Y	U	Y	Y	U	N	N	Y	7	Moderate risk
**Passmore et al.2006**	Y	Y	Y	Y	U	Y	Y	N	N	N	Y	7	Moderate risk
**Saidu et al.2021**	Y	Y	Y	U	U	Y	Y	Y	N	N	Y	7	Moderate risk
**Segondy et al. 2016**	Y	Y	Y	U	N	Y	Y	Y	U	U	N	6	Moderate risk
**Snyman et al. 2023**	Y	Y	Y	Y	U	Y	Y	N	N	N	Y	7	Moderate risk
**Sørbye et al.2023**	Y	Y	Y	Y	U	Y	Y	N	N	N	Y	7	Moderate risk
**Tayib et al.2015**	Y	Y	Y	Y	Y	Y	Y	Y	N	N	Y	9	Low risk
**Taylor et al.2011**	Y	Y	Y	U	U	Y	Y	Y	N	N	Y	7	Moderate risk
**Van Aardt et al.2016**	Y	Y	Y	Y	Y	Y	Y	Y	N	N	Y	9	Low risk
**Van Aardt et al.2015**	Y	Y	Y	U	N	Y	Y	N/A	N	N	Y	6	Moderate risk
**Vink et al.2023**	Y	Y	Y	U	N	Y	Y	N	N	N	Y	6	Moderate risk

**Wang et al.2011**	Y	Y	Y	Y	Y	Y	Y	Y	N	N	Y	9	Low risk
**Zeier et al.2015**	Y	Y	Y	Y	Y	Y	Y	Y	N	N	Y	9	Low risk

Y: Yes N: No N/A: Not applicable U: Unclear

Q1. Were the two groups similar and recruited from the same population?
Q2. Were the exposures measured similarly to assign people to both exposed and unexposed groups?
Q3. Was the exposure measured in a valid and reliable way?
Q4. Were confounding factors identified?
Q5. Were strategies to deal with confounding factors stated?
Q6. Were the groups/participants free of the outcome at the start of the study (or at the moment of exposure)?
Q7. Were the outcomes measured in a valid and reliable way?
Q8. Was the follow up time reported and sufficient to be long enough for outcomes to occur?
Q9. Was follow up complete, and if not, were the reasons to loss to follow up described and explored?
Q10. Were strategies to address incomplete follow up utilized?
Q11. Was appropriate statistical analysis used?

Table S4: Case-control

**Study ID**	**Q1**	**Q2**	**Q3**	**Q4**	**Q5**	**Q6**	**Q7**	**Q8**	**Q9**	**Q10**	**Total**	**Overall Judgment**
**Allan et al. 2006**	Y	Y	Y	Y	Y	U	U	Y	U	Y	7	Moderate risk
**Allan et al. 2008**	Y	Y	Y	Y	Y	Y	U	Y	U	U	7	Moderate risk
**Cooper et al. 1992**	N	U	U	Y	Y	N/A	N/A	Y	N/A	U	3	High risk
**De Vuyst et al. 2012**	Y	U	Y	Y	Y	Y	Y	Y	N	Y	8	Low risk
**Kay et al. 2005**	Y	Y	Y	Y	Y	U	U	Y	U	Y	7	Moderate risk
**Marais et al. 2008**	Y	Y	Y	Y	Y	Y	Y	U	U	Y	8	Low risk
**Sebitloane et al. 2024**	Y	U	Y	Y	Y	Y	N	Y	Y	Y	8	Low risk
**Tylor et al. 2010**	Y	U	Y	Y	Y	Y	Y	Y	Y	Y	8	Low risk

**Van de Wijgert et al.2020**	Y	Y	Y	Y	Y	Y	Y	Y	Y	Y	10	Low risk

Y: Yes N: No N/A: Not applicable U: Unclear

Q.1 Were the groups comparable other than presence of disease in cases or absence of disease in controls?
Q2. Were cases and controls matched appropriately?
Q3. Were the same criteria used for identification of cases and controls?
Q4. Was exposure measured in a standard, valid and reliable way?
Q5. Was exposure measured in the same way for cases and controls?
Q6. Were confounding factors identified?
Q7. Were strategies to deal with confounding factors stated?
Q8. Were outcomes assessed in a standard, valid and reliable way for cases and controls?
Q9. Was the exposure period of interest long enough to be meaningful?
Q10. Was appropriate statistical analysis used?

Table S5: Case series

Study ID	Q1	Q2	Q3	Q4	Q5	Q6	Q7	Q8	Q9	Q10	Total	Overall Judgement
**Grayson et al. 1996**	U	Y	Y	U	U	Y	Y	U	N	U	4	High risk
**Johnson et al.1991**	Y	Y	Y	U	Y	U	Y	N	N	N	5	High risk
**Mbatani et al.2016**	Y	Y	Y	Y	Y	Y	Y	Y	N	Y	9	Low risk
**Padayachee et al.1993**	Y	Y	Y	U	U	N	Y	Y	N	N	5	High risk
**Rad et al. 2017**	Y	Y	Y	U	Y	Y	Y	Y	N	N	7	Moderate risk
**Wright et al. 1995**	Y	Y	Y	U	Y	Y	Y	N	Y	N	7	Moderate risk

Y: Yes N: No N/A: Not applicable U: Unclear

Q1. Were there clear criteria for inclusion in the case series?
Q2. Was the condition measured in a standard, reliable way for all participants included in the case series?
Q3. Were valid methods used for identification of the condition for all participants included in the case series?
Q4. Did the case series have consecutive inclusion of participants?
Q5. Did the case series have complete inclusion of participants?

Q6. Was there clear reporting of the demographics of the participants in the study?
Q7. Was there clear reporting of clinical information of the participants?
Q8. Were the outcomes or follow-up results of cases clearly reported?
Q9. Was there clear reporting of the presenting site(s)/clinic(s) demographic information?
Q10 Was statistical analysis appropriate?
