# Supplementary material for: Mapping the HPV Landscape in South African Women: A Systematic Review and Meta-Analysis of Viral Genotypes, Microbiota, and Immune Signals
Source: Viruses. 2024 Dec 8;16(12):1893. doi: 10.3390/v16121893 (PMC11680443; doi:10.3390/v16121893)
Supplement: Supplementary file 1 [file viruses-16-01893-s001.zip › Table S1. Reasons for excluding studies.pdf]

Supplementary Material SB.

Table S1. Reasons for excluding studies from the systematic review after reading full-text of the studies

NO.	Studies excluded from the systematic review	Reasons for exclusion
1	Abud-Mendoza, C., et al., *Decreased blood levels of B lymphocytes and NK cells in patients with systemic lupus erythematosus (SLE) infected with papillomavirus (HPV).* Archives of Dermatological Research, 2013. **305**(2): p. 117-123. DOI: 10.1007/s00403-012-1258-9.	Not geographically relevant
2	Achdiat, P.A., et al., *Vulvar squamous cell carcinoma due to human papillomavirus type 11.* Skin Research and Technology, 2023. **29**(8). DOI: 10.1111/srt.13436.	Letter to editor
3	Adebamowo, S.N., et al., *Persistent Low-Risk and High-Risk Human Papillomavirus Infections of the Uterine Cervix in HIV-Negative and HIV-Positive Women.* FRONTIERS IN PUBLIC HEALTH, 2017. **5**. DOI: 10.3389/fpubh.2017.00178.	Not geographically relevant
4	Adler, D., et al., *Human papillomavirus genotype distribution among human immunodeficiency virus (HIV)-infected and non-HIV-infected women in Soweto, South Africa.* Journal of Clinical Microbiology, 2008. **46**(12): p. 4109-4110. DOI: 10.1128/JCM.01667-08.	Letter to editor
5	Adler, D.H., et al., *High Level of Agreement between Clinician-Collected and Self-Collected Samples for HPV Detection among South African Adolescents.* Journal of Pediatric and Adolescent Gynecology, 2012. **25**(4): p. 280-281. DOI: 10.1016/j.jpag.2012.04.006.	Letter to editor
6	Adler, D.H., et al., *Increased regression and decreased incidence of human papillomavirus-related cervical lesions among HIV-infected women on HAART.* AIDS, 2012. **26**(13): p. 1645-1652. DOI: 10.1097/QAD.0b013e32835536a3.	No HPV prevalence data reported
7	Al Moustafa, A.E., et al., *High-risk human papillomaviruses and epstein-barr virus presence and crosstalk in human oral carcinogenesis*, in *Development of Oral Cancer: Risk Factors and Prevention Strategies*. 2017, Springer International Publishing. p. 83-94. DOI: 10.1007/978-3-319-48054-1_6.	Not geographically relevant
8	Ali, K.S., H.Y.M. Ali, and J.M.S. Jubrael, *Concentration levels of IL-10 and TNFα cytokines in patients with human papilloma virus (HPV) DNA+ and DNA- cervical lesions.* Journal of Immunotoxicology, 2012. **9**(2): p. 168-172. DOI: 10.3109/1547691X.2011.642419.	Not geographically relevant

9	Amaral, C.M.M., et al., *MDM2 polymorphism associated with the development of cervical lesions in women infected with Human papillomavirus and using of oral contraceptives.* Infectious Agents and Cancer, 2014. **9**(1). DOI: 10.1186/1750-9378-9-24.	Not geographically relevant
10	Anderson, E.L., et al., *Human Papillomavirus Type 16 L2 DNA Methylation in Exfoliated Cervical Cells from College-Age Women.* Journal of Lower Genital Tract Disease, 2016. **20**(4): p. 332-337. DOI: 10.1097/QAI.0b013e3181b7354c.	Not geographically relevant
11	Annunziata, C., et al., *Prevalence of "unclassified" HPV genotypes among women with abnormal cytology.* Infectious Agents and Cancer, 2018. **13**(1). DOI: 10.1186/s13027-018-0199-0.	Not geographically relevant
12	Audirac-Chalifour, A., et al., *Cervical microbiome and cytokine profile at various stages of cervical cancer: A pilot study.* PLoS ONE, 2016. **11**(4). DOI: 10.1371/journal.pone.0153274.	Not geographically relevant
13	Austin, R.M., A.B. Olawaiye, and C. Zhao, *Significance of Human Papillomavirus Test Performance in Samples from Women with Histopathologically Confirmed Invasive Cervical Cancer.* Journal of Lower Genital Tract Disease, 2016. **20**(3): p. e52. DOI: 10.1097/LGT.0000000000000218.	Letter to editor
14	Badial, R.M., et al., *Detection and genotyping of human papillomavirus (HPV) in HIV-infected women and its relationship with HPV/HIV co-infection.* Medicine (United States), 2018. **97**(14). DOI: 10.1097/MD.0000000000009545.	Not geographically relevant
15	Ball, S.L., et al., *Analyses of human papillomavirus genotypes and viral loads in anogenital warts.* Journal of Medical Virology, 2011. **83**(8): p. 1345-1350. DOI: 10.1002/jmv.22111.	Not geographically relevant
16	Barbisan, G., et al., *TNF-α and IL-10 promoter polymorphisms, HPV infection, and cervical cancer risk.* Tumor Biology, 2012. **33**(5): p. 1549-1556. DOI: 10.1007/s13277-012-0408-.	Not geographically relevant
17	Bellizzi, A.M., et al., *Basaloid squamous cell carcinoma of the esophagus: Assessment for high-risk human papillomavirus and related molecular markers.* American Journal of Surgical Pathology, 2009. **33**(11): p. 1608-1614. DOI: 10.1097/PAS.0b013e3181b46fd4.	Location not specified
18	Berggrund, M., et al., *Temporal changes in the vaginal microbiota in self-samples and its association with persistent HPV16 infection and CIN2+.* Virology Journal, 2020. **17**(1). DOI: 10.1186/s12985-020-01420-z.	Not geographically relevant
19	Branca, M., et al., *Factors predicting the persistence of genital human papillomavirus infections and PAP smear abnormality in HIV-positive and HIV-*	Not geographically relevant

	negative women during prospective follow-up. International Journal of STD and AIDS, 2003. **14**(6): p. 417-425. DOI: 10.1258/095646203765371321.	
20	Brito, M.J., et al., *Detection of HIV mRNA in routine liquid-based cytology specimens of HIV-infected women.* Cytopathology, 2021. **32**(5): p. 640-645. DOI: 10.1111/cyt.12983.	Not geographically relevant
21	Bulane, A., et al., *Human papillomavirus DNA in head and neck squamous cell carcinomas in the Free State, South Africa.* Journal of Medical Virology, 2020. **92**(2): p. 227-233. DOI: 10.1002/jmv.25556.	HPV data not separated based on gender
22	Bulk, S., et al., *The contribution of HPV18 to cervical cancer is underestimated using high-grade CIN as a measure of screening efficiency.* British Journal of Cancer, 2007. **96**(8): p. 1234-1236. DOI: 10.1038/sj.bjc.6603693.	Not geographically relevant
23	Caixeta, R.C.A., et al., *Association between the human papillomavirus, bacterial vaginosis and cervicitis and the detection of abnormalities in cervical smears from teenage girls and young women.* Diagnostic Cytopathology, 2015. **43**(10): p. 780-785. DOI: 10.1002/dc.23301.	Not geographically relevant
24	Camargo, M., et al., *Association of HIV status with infection by multiple HPV types.* Tropical Medicine and International Health, 2018. **23**(11): p. 1259-1268. DOI: 10.1111/tmi.13142.	Not geographically relevant
25	Carr, N.J., et al., *Squamous cell papillomas of the esophagus: A study of 23 lesions for human papillomavirus by in situ hybridization and the polymerase chain reaction.* Human Pathology, 1994. **25**(5): p. 536-540. DOI: 10.1016/0046-8177(94)90128-7.	Not geographically relevant
26	Chávez-Torres, M., et al., *The vaginal microbiota of women living with HIV on suppressive antiretroviral therapy and its relation to high-risk human papillomavirus infection.* BMC Microbiology, 2023. **23**(1). DOI: 10.1186/s12866-023-02769-1.	Not geographically relevant
27	Chambuso, R., et al., *Human Leukocyte Antigen (HLA) Class II-DRB1 and-DQB1 Alleles and the Association with Cervical Cancer in HIV/HPV Co-Infected Women in South Africa.* JOURNAL OF CANCER, 2019. **10**(10): p. 2145-2152. DOI: 10.7150/jca.25600	No HPV prevalence data reported
28	Chowdhury, S., et al., *HPV Type Distribution in Benign, High-Grade Squamous Intraepithelial Lesions and Squamous Cell Cancers of the Anus by HIV Status.* Cancers, 2023. **15**(3). DOI: 10.3390/cancers15030660.	Location not specified
29	Collins, S., et al., *Cigarette smoking is an independent risk factor for cervical intraepithelial neoplasia in young women: A longitudinal study.* European Journal of Cancer, 2010. **46**(2): p. 405-411. DOI: 10.1016/j.ejca.2009.09.015.	Not geographically relevant

30	Combrinck, C.E., R.Y. Seedat, and F.J. Burt, *FRET-based detection and genotyping of HPV-6 and HPV-11 causing recurrent respiratory papillomatosis.* Journal of Virological Methods, 2013. **189**(2): p. 271-276. DOI: 10.1016/j.jviromet.2013.01.025.	HPV data not separated based on gender
31	Combrinck, C.E., et al., *Novel HPV-6 variants of human papillomavirus causing recurrent respiratory papillomatosis in southern Africa.* Epidemiology and Infection, 2012. **140**(6): p. 1095-1101. DOI: 10.1017/S0950268811001580.	HPV data not separated based on gender
32	Cooper, K., et al., *Episomal and integrated human papillomavirus in cervical neoplasia shown by non-isotopic in situ hybridisation.* Journal of Clinical Pathology, 1991. **44**(12): p. 990-996. DOI: 10.1136/jcp.44.12.990.	Location not specified
33	Cooper, K., *Human papillomavirus and endocervical adenocarcinoma.* Human Pathology, 1994. **25**(2): p. 216-217. DOI: 10.1016/0046-8177(94)90288-7.	Letter to editor
34	Cooper, K., *Physical state of human papillomavirus using non-isotopic in situ hybridization.* Journal of Clinical Pathology, 1995. **48**(8): p. 786-787. DOI: 10.1136/jcp.48.8.786.	Letter to editor
35	Cooper, K., Z. Haffajee, and L. Taylor, *Human papillomavirus and schistosomiasis associated bladder cancer.* Journal of Clinical Pathology - Molecular Pathology, 1997. **50**(3): p. 145-148. DOI: 10.1136/mp.50.3.145.	Gender not specified
36	Cooper, K., et al., Labelling pattern obtained by non-isotopic in situ hybridization as a prognostic factor in HPV-associated lesions (multiple letters) [1]. Journal of Pathology, 1997. 182(3): p. 367. DOI: 10.1002/(SICI)1096-9896(199707)182:3<367::AID-PATH844>3.0.CO;2-P.	Letter to editor
37	Cooper, K. and J. McGee, *HPV genotypes in cervical neoplasia in South Africa [2].* Journal of Clinical Pathology, 1992. **45**(1): p. 90. DOI: 10.1136/jcp.45.1.90-a. DOI: 10.1136/jcp.45.1.90-a.	Letter to editor
38	da Mota Vasconcelos Brasil, C., C.M.B. Ribeiro, and J.C. Leão, *Oral and Genital Human Herpesvirus 8 and Human Papillomavirus in heterosexual partners.* Journal of Oral Pathology and Medicine, 2013. **42**(1): p. 61-65. DOI: 10.1111/j.1600-0714.2012.01184.x.	Not geographically relevant
39	De Castro-Sobrinho, J.M., et al., *Bacterial vaginosis and inflammatory response showed association with severity of cervical neoplasia in HPV-positive women.* Diagnostic Cytopathology, 2016. **44**(2): p. 80-86. DOI: 10.1002/dc.23388.	Letter to editor
40	Deleré, Y., et al., *Cervicovaginal self-sampling is a reliable method for determination of prevalence of human papillomavirus genotypes in women aged 20 to 30 years.* Journal of Clinical Microbiology, 2011. **49**(10): p. 3519-3522. DOI: 10.1128/JCM.01026-11.	Not geographically relevant

41	Denny, L.A. and T.C. Wright Jr, *Human papillomavirus testing and screening.* Best Practice and Research: Clinical Obstetrics and Gynaecology, 2005. **19**(4 SPEC. ISS.): p. 501-515. DOI: 10.1016/j.bpobgyn.2005.02.004.	Non-original research publications
42	Denny, L., *Human papillomavirus infections: Epidemiology, clinical aspects and vaccines.* Open Infectious Diseases Journal, 2009. **3**(SPEC.ISS.1): p. 135-142. DOI: 10.2174/1874279301004010135.	Non-original research publications
43	Denny, L., *Nine-valent human papillomavirus vaccine: great science, but will it save lives?* The Lancet, 2017. **390**(10108): p. 2123-2124. DOI: 10.1016/S0140-6736(17)32144-X.	Location not specified
44	Denny, L., *Human papilloma virus in gynaecology.* Best Practice and Research: Clinical Obstetrics and Gynaecology, 2018. **47**: p. 1. DOI: 10.1016/j.bpobgyn.2017.11.006.	Non-original research publications
45	Denny, L., et al., *Performance of an Human Papillomavirus Test in Samples from Women with Histopathologically Confirmed Invasive Cervical Cancer.* Journal of Lower Genital Tract Disease, 2016. **20**(3): p. e52-e53. DOI: 10.1097/LGT.0000000000000228.	Non-original research publications
46	Di Paola, M., et al., *Characterization of cervico-vaginal microbiota in women developing persistent high-risk Human Papillomavirus infection.* Scientific Reports, 2017. **7**(1). DOI: 10.1038/s41598-017-09842-6.	Not geographically relevant
47	Dikkers, F.G., R.E.A. Tjon Pian Gi, and M.R.M. San Giorgi, *Recurrent respiratory papillomatosis and human papillomavirus*, in *Infections of the Ears, Nose, Throat, and Sinuses*. 2018, Springer International Publishing. p. 365-376. DOI 10.1007/978-3-319-74835-1_29.	Non-original research publications
48	Donà, M.G., et al., *Human papillomavirus detection in matched oral rinses, oropharyngeal and oral brushings of cancer-free high-risk individuals.* Oral Oncology, 2019. **91**: p. 1-6. DOI: 10.1016/j.oraloncology.2019.02.002.	Not geographically relevant
49	Douglas, J.M. and E.R. Unger, *Genital Human Papillomavirus Infections*, in *Atlas of Sexually Transmitted Diseases and AIDS, Fourth Edition*. 2010, Elsevier. p. 186-202. DOI 10.1016/B978-0-7020-4060-3.00011-9.	Non-original research publications
50	Dreyer, G., C. Maske, and M. Stander, *Clinical evaluation and budget impact analysis of cervical cancer screening using cobas 4800 HPV screening technology in the public sector of South Africa.* PLOS ONE, 2019. **14**(9). DOI: 10.1371/journal.pone.0221495.	No HPV prevalence data reported
51	Du, P., *Human papillomavirus infection and cervical cancer in HIV+ women*, in *Cancer Treatment and Research*. 2019, Springer International Publishing. p. 105-129. DOI 10.1007/978-3-030-03502-0_5.	Non-original research publications

52	Dube Mandishora, R.S., et al., *Intra-host sequence variability in human papillomavirus.* Papillomavirus Research, 2018. **5**: p. 180-191. DOI: 10.1016/j.pvr.2018.04.006.	Not geographically relevant
53	Fatahzadeh, M., et al., *Oral human papillomavirus detection in older adults who have human immunodeficiency virus infection.* Oral Surgery, Oral Medicine, Oral Pathology and Oral Radiology, 2013. **115**(4): p. 505-514. DOI: 10.1016/j.oooo.2012.11.004.	Not geographically relevant
54	Firnhaber, C., et al., *Cryotherapy Reduces Progression of Cervical Intraepithelial Neoplasia Grade 1 in South African HIV-Infected Women: A Randomized, Controlled Trial.* JAIDS-JOURNAL OF ACQUIRED IMMUNE DEFICIENCY SYNDROMES, 2017. **76**(5): p. 532-538. DOI: 10.1097/QAI.0000000000001539.	No HPV prevalence data reported
55	Firnhaber, C., et al., *Human Papillomavirus Vaccination Prior to Loop Electroexcision Procedure Does Not Prevent Recurrent Cervical High-grade Squamous Intraepithelial Lesions in Women Living With Human Immunodeficiency Virus: A Randomized, Double-blind, Placebo-controlled Trial.* CLINICAL INFECTIOUS DISEASES, 2021. **73**(7): p. E2211-E2216. DOI: 10.1093/cid/ciaa1456.	No HPV prevalence data reported
56	Formánek, M., et al., *Laryngopharyngeal Reflux Is a Potential Risk Factor for Juvenile-Onset Recurrent Respiratory Papillomatosis.* BioMed Research International, 2019. **2019**. DOI: 10.1155/2019/1463896.	Location not specified
57	Fujii, T., et al., *Comparison between in situ hybridization and real-time PCR technique as a means of detecting the integrated form of human papillomavirus 16 in cervical neoplasia.* Diagnostic Molecular Pathology, 2005. **14**(2): p. 103-108. DOI: 10.1097/01.pas.0000162755.84026.9f.	Not geographically relevant
58	Galati, L., et al., *Detection of Circulating HPV16 DNA as a Biomarker for Cervical Cancer by a Bead-Based HPV Genotyping Assay.* Microbiology Spectrum, 2022. **10**(2). DOI: 10.1128/spectrum.01480-21.	Not geographically relevant
59	Gangkofner, D.S., et al., *Patterns of antibody responses to nonviral cancer antigens in head and neck squamous cell carcinoma patients differ by human papillomavirus status.* International Journal of Cancer, 2019. **145**(12): p. 3436-3444. DOI: 10.1002/ijc.32623.	Not geographically relevant
60	Garlick, J.A., et al., *Detection of human papillomavirus (HPV) DNA in focal epithelial hyperplasia.* Journal of Oral Pathology & Medicine, 1989. **18**(3): p. 172-177. DOI: 10.1111/j.1600-0714.1989.tb00757.x.	Location not specified

61	Gómez, F., et al., *Labelling pattern obtained by non-isotopic in situ hybridization as a prognostic factor in HPV-associated lesions.* Journal of Pathology, 1996. **179**(3): p. 272-275. DOI: 10.1002/(SICI)1096-9896(199607)179:3<272::AID-PATH599>3.0.CO;2-E.	Location not specified
62	Govan, V.A., et al., *Ethnic differences in allelic distribution of IFN-g in South African women but no link with cervical cancer.* Journal of Carcinogenesis, 2003. **2**. DOI: 10.1186/1477-3163-2-3.	No HPV prevalence data reported
63	Govan, V.A., et al., *The allelic distribution of-308 Tumor Necrosis Factor-alpha gene polymorphism in South African women with cervical cancer and control women.* BMC CANCER, 2006. **6**. DOI: 10.1186/1471-2407-6-24.	No HPV prevalence data reported
64	Grayson, W., L.F. Taylor, and K. Cooper, *Carcinosarcoma of the uterine cervix : A report of eight cases with immunohistochemical analysis and evaluation of human papillomavirus status.* American Journal of Surgical Pathology, 2001. **25**(3): p. 338-347. DOI: 10.1097/00000478-200103000-00008.	Location not specified
65	Grayson, W., L.F. Taylor, and K. Cooper, *Carcinosarcoma of the uterine cervix : A report of eight cases with immunohistochemical analysis and evaluation of human papillomavirus status.* American Journal of Surgical Pathology, 2001. **25**(3): p. 338-347. DOI: 10.1136/jcp.55.2.108.	Location not specified
66	Guo, Y.L., et al., *Bacterial vaginosis is conducive to the persistence of HPV infection.* International Journal of STD and AIDS, 2012. **23**(8): p. 581-584. DOI: 10.1258/ijsa.2012.011342.	Not geographically relevant
67	Gupta, R., et al., *High Prevalence of Cervical High-Grade Lesions and High-Risk Human Papillomavirus Infections in Women Living with HIV: A Case for Prioritizing Cervical Screening in This Vulnerable Group.* Acta Cytologica, 2022. **66**(6): p. 496-506. DOI: 10.1159/000525340.	Location not specified
68	Gustavsson, I., et al., *Clinical validation of the HPVIR high-risk HPV test on cervical samples according to the international guidelines for human papillomavirus DNA test requirements for cervical cancer screening.* Virology Journal, 2019. **16**(1). DOI: 10.1186/s12985-019-1216-7.	Not geographically relevant
69	Harris, T.G., et al., *Depot-medroxyprogesterone acetate and combined oral contraceptive use and cervical neoplasia among women with oncogenic human papillomavirus infection.* American Journal of Obstetrics and Gynecology, 2009. **200**(5): p. 489.e1-489.e8. DOI: 10.1016/j.ajog.2009.01.030.	Not geographically relevant
70	Hasanzadeh, M., et al., *The interaction of high and low-risk human papillomavirus genotypes increases the risk of developing genital warts: A population-based*	Not geographically relevant

	cohort study. Journal of Cellular Biochemistry, 2019. **120**(8): p. 12870-12874. DOI: 10.1002/jcb.28557.	
71	Henke, R.P., et al., *Human papillomavirus type 13 and focal epithelial hyperplasia of the oral mucosa: DNA hybridization on paraffin-embedded specimens.* Virchows Archiv A Pathological Anatomy and Histopathology, 1987. **411**(2): p. 193-198. DOI: 10.1007/BF00712744.	Not geographically relevant
72	Herrington, C.S., et al., *Detection of high risk human papillomavirus in routine cervical smears: Strategy for screening.* Journal of Clinical Pathology, 1992. **45**(5): p. 385-390. DOI: 10.1136/jcp.45.5.385.	Not geographically relevant
73	Herrington, C.S., et al., *Screening For High- and Low-Risk Human Papillomavirus Types In Single Routine Cervical Smears By Non-Isotopic In Situ Hybridization.* Cytopathology, 1992. **3**(2): p. 71-78. DOI: 10.1111/j.1365-2303.1992.tb00028.x.	Not geographically relevant
74	Herrington, C.S., et al., *Morphological correlation of human papillomavirus infection of matched cervical smears and biopsies from patients with persistent mild cervical cytological abnormalities.* Human Pathology, 1995. **26**(9): p. 951-955. DOI: 10.1016/0046-8177(95)90083-7.	Not geographically relevant
75	Herrington, C.S., et al., *Human papillomavirus status in the prediction of high-grade cervical intraepithelial neoplasia in patients with persistent low-grade cervical cytological abnormalities.* British Journal of Cancer, 1995. **71**(1): p. 206-209. DOI: 10.1038/bjc.1995.42.	Location not specified
76	Hidalgo-Tenorio, C., et al., *HPV Infection of the Oropharyngeal, Genital and Anal Mucosa and Associated Dysplasia in People Living with HIV.* Viruses, 2023. **15**(5). DOI: 10.3390/v15051170.	Not geographically relevant
77	Huang, X., et al., *Cervicovaginal microbiota composition correlates with the acquisition of high-risk human papillomavirus types.* International Journal of Cancer, 2018. **143**(3): p. 621-634. DOI: 10.1002/ijc.31342.	Not geographically relevant
78	Husseinzadeh, N., et al., *HPV changes and their significance in patients with invasive squamous cell carcinoma of the vulva: A clinicopathologic study.* Gynecologic Oncology, 1991. **43**(3): p. 237-241. DOI: 10.1016/0090-8258(91)90027-3.	Not geographically relevant
79	Hwang, L.Y. and A.B. Moscicki, *Human Papilloma Virus Infection in Women*, in *Women and Health, Second Edition*. 2012, Elsevier. p. 523-534. DOI 10.1016/B978-0-12-384978-6.00034-0.	Non-original research publications

	Izaaks, C.D., E.J. Truter, and S. Khan, *Prevalence of human papilloma virus in cytological abnormalities: Association of risk factors and cytomorphological findings.* CytoJournal, 2012. **9**(1). DOI: 10.4103/1742-6413.100123.	Not geographically relevant
80	Jain, B., et al., *Human papilloma virus infection of uterine cervix and spectrum of cervical pathology in human immunodeficiency virus/AIDS.* Journal of Cancer Research and Therapeutics, 2021. **17**(6): p. 1462-1467. DOI: 10.4103/jcrt.JCRT_552_19.	Location not specified
81	Jones, J., et al., *Comparison of the PapilloCheck® DNA micro-array Human Papillomavirus detection assay with Hybrid Capture II and PCR-enzyme immunoassay using the GP5/6+ primer set.* Journal of Clinical Virology, 2009. **45**(2): p. 100-104. DOI: 10.1016/j.jcv.2009.02.013.	Not geographically relevant
82	Kaelin, E.A., et al., *Cervicovaginal DNA Virome Alterations Are Associated with Genital Inflammation and Microbiota Composition.* mSystems, 2022. **7**(2). DOI: 10.1128/msystems.00064-22.	Not geographically relevant
83	Kahn, J.A., et al., *Psychological, behavioral, and interpersonal impact of human papillomavirus and pap test results.* Journal of Women's Health, 2005. **14**(7): p. 650-659. DOI: 10.1089/jwh.2005.14.650.	Not geographically relevant
84	Kasap, B., et al., *Prevalence and risk factors for human papillomavirus DNA in cervical cytology.* European Journal of Obstetrics and Gynecology and Reproductive Biology, 2011. **159**(1): p. 168-171. DOI: 10.1016/j.ejogrb.2011.06.021.	HPV data not separated based on country
85	Khanna, N., et al., *Human papillomavirus detection in self-collected vaginal specimens and matched clinician-collected cervical specimens.* International Journal of Gynecological Cancer, 2007. **17**(3): p. 615-622.	Not geographically relevant
86	Kremer, W.W., et al., *The use of molecular markers for cervical screening of women living with HIV in South Africa.* AIDS, 2019. **33**(13): p. 2035-2042. DOI: 10.1097/QAD.0000000000002325.	No HPV prevalence data reported
87	Kuhn, L., R. Saidu, and L. Denny, *Assessing the diagnostic value of Xpert HPV – Authors' reply.* The Lancet Global Health, 2020. **8**(8): p. e998. DOI: 10.1016/S2214-109X(20)30296-5.	Non-original research publications
88	Kýký, Y., et al., *Detection of human papillomavirus infection in esophageal carcinomas by the histopathological method and polymerase chain reaction technique.* Turkish Journal of Medical Sciences, 2002. **32**(3): p. 223-230.	Not geographically relevant

89	Lauricella-Lefebvre, M.A., et al., *High rate of multiple genital HPV infections detected by DNA hybridization.* Journal of Medical Virology, 1992. **36**(4): p. 265-270. DOI: 10.1002/jmv.1890360406.	Not geographically relevant
	Lavergne, D. and E.M. De Villiers, *Papillomavirus in esophageal papillomas and carcinomas.* International Journal of Cancer, 1999. **80**(5): p. 681-684. DOI: 10.1002/(SICI)1097-0215(19990301)80:5<681::AID-IJC8>3.0.CO;2-A.	HPV data not separated based on gender
	Louvanto, K., et al., *Role of Human Leukocyte Antigen Allele Sharing in Human Papillomavirus Infection Transmission Among Heterosexual Couples: Findings From the HITCH Cohort Study.* Journal of Infectious Diseases, 2022. **226**(7): p. 1175-1183. DOI: 10.1093/infdis/jiac115.	Not geographically relevant
	Luo, Q., et al., *Epidemiologic characteristics of high-risk HPV and the correlation between multiple infections and cervical lesions.* BMC Infectious Diseases, 2023. **23**(1). DOI: 10.1186/s12879-023-08634-w.	Not geographically relevant
90	Malin, K., et al., *Optimization of droplet digital PCR assays for the type-specific detection and quantification of five HPV genotypes, including additional data on viral loads of nine different HPV genotypes in cervical carcinomas.* Journal of Virological Methods, 2021. **294**. DOI: 10.1016/j.jviromet.2021.114193.	Not geographically relevant
91	Marais, D., et al., *The effectiveness of Carraguard, a vaginal microbicide, in protecting women against high-risk human papillomavirus infection.* Antiviral Therapy, 2011. **16**(8): p. 1219-1226. DOI: 10.3851/IMP1890.	No HPV prevalence data reported
92	Matsha, T., et al., *Human papillomavirus associated with oesophageal cancer.* Journal of Clinical Pathology, 2002. **55**(8): p. 587-590. DOI: 10.1136/jcp.55.8.587.	HPV data not separated based on gender
93	Matsukura, T. and M. Sugase, *Identification of genital human papillomaviruses in cervical biopsy specimens: Segregation of specific virus types in specific clinicopathologic lesions.* International Journal of Cancer, 1995. **61**(1): p. 13-22. DOI: 10.1002/ijc.2910610104.	Location not specified
94	Mboumba Bouassa, R.S., et al., *High Prevalence of Cervical High-Risk Human Papillomavirus Harboring Atypical Genotypes in Human Immunodeficiency Virus -Infected and -Uninfected First-Generation Adult Immigrant Women Originating from Sub-Saharan Africa and Living in France.* Journal of Immigrant and Minority Health, 2021. **23**(2): p. 308-319. DOI: 10.1007/s10903-020-01074-7.	HPV data not separated based on country
95	Mbulawa, Z.Z.A., et al., *The impact of human immunodeficiency virus on human papillomavirus transmission in heterosexually active couples.* JOURNAL OF INFECTION, 2013. **67**(1): p. 51-58. DOI: 10.1016/j.jinf.2013.03.009.	No HPV prevalence data reported

96	Meisels, A. and C. Morin, *Human papillomavirus and cancer of the uterine cervix.* Gynecologic Oncology, 1981. **12**(2 PART 1): p. S111-S123. DOI: 10.1016/0090-8258(81)90066-4.	Not geographically relevant
97	Menezes, L.J., et al., *Patterns of prevalent HPV and STI co-infections and associated factors among HIV-negative young Western Cape, South African women: The EVRI trial.* Sexually Transmitted Infections, 2018. **94**(1): p. 55-61.	No HPV prevalence data reported
98	Mhatre, M., et al., *Cervical intraepithelial neoplasia is associated with genital tract mucosal inflammation.* Sexually Transmitted Diseases, 2012. **39**(8): p. 591-597. DOI: 10.1097/OLQ.0b013e318255aeef.	Not geographically relevant
99	Moodley, J.R., et al., HIV and pre-neoplastic and neoplastic lesions of the cervix in South Africa: a case-control study. BMC CANCER, 2006. 6. DOI: 10.1186/1471-2407-6-135	No HPV prevalence data reported
100	Moscicki, A.B., et al., *Redetection of cervical human papillomavirus type 16 (HPV16) in women with a history of HPV16.* Journal of Infectious Diseases, 2013. **208**(3): p. 403-412. DOI: 10.1093/infdis/jit175.	Not geographically relevant
101	Moselhi, M., et al., *Human papillomavirus detection to screen for cervical cancer.* Journal of the American Medical Association, 2000. **284**(1): p. 39-40. DOI:10.1001/jama.284.1.39.	Non-original research publications
102	Mumba, E., et al., *Human papillomaviruses do not play an aetiological role in Müllerian adenosarcomas of the uterine cervix.* Journal of Clinical Pathology, 2008. **61**(9): p. 1041-1044. DOI: 10.1136/jcp.2008.056614.	HPV data not separated based on gender
103	Muñoz-Hernando, L., et al., *Prevalence of Anal High-Risk Human Papilloma Virus Infection and Abnormal Anal Cytology among Women Living with HIV.* Journal of Personalized Medicine, 2022. **12**(11). DOI: 10.3390/jpm12111778.	Not geographically relevant
104	Narasimhan, M., et al., *The case for integrated human papillomavirus vaccine and HIV prevention with broader sexual and reproductive health and rights services for adolescent girls and young women.* Transactions of the Royal Society of Tropical Medicine and Hygiene, 2017. **111**(4): p. 141-143. DOI: 10.1093/trstmh/trx032.	Non-original research publications
105	Nilyanimit, P., et al., *Comparison of four human papillomavirus genotyping methods: Next-generation sequencing, INNO-LiPA, electrochemical DNA Chip, and nested-PCR.* Annals of Laboratory Medicine, 2018. **38**(2): p. 139-146. DOI: 10.3343/alm.2018.38.2.139.	Not geographically relevant
106	Nonato, D.R., et al., *Prevalence and factors associated with coinfection of human papillomavirus and Chlamydia trachomatis in adolescents and young women.* American Journal of Obstetrics and Gynecology, 2016. **215**(6): p. 753.e1-753.e9. DOI: 10.1016/j.ajog.2016.07.003.	Not geographically relevant

107	Odendaal, L.N., et al., *The prevalence of human papillomavirus in ocular surface squamous neoplasia in HIV positive and negative patients in a South African population.* Infection, 2024. **52**(4): p. 1547-1552. DOI: 10.1007/s15010-024-02289-8.	HPV data not separated based on gender
108	Olukomogbon, T., et al., *Association Between Cervical Inflammatory Mediators and Prevalent Cervical Human Papillomavirus Infection.* JCO Global Oncology, 2024. **10**. DOI: 10.1200/GO.23.00380.	Not geographically relevant
109	Padalko, E., et al., *Prospective evaluation of E6/E7 mRNA detection by the NucliSENS Easy Q HPV assay in a stepwise protocol.* Journal of Medical Virology, 2013. **85**(7): p. 1242-1249. DOI: 10.1002/jmv.23591.	Not geographically relevant
110	Padayachee, A. and C.W. Van Wyk, *Human papillomavirus (HPV) in oral squamous cell papillomas.* Journal of Oral Pathology & Medicine, 1987. **16**(7): p. 353-355. DOI: 10.1111/j.1600-0714.1987.tb00707.x.	Location not specified
111	Palazzi, M.A., et al., *Detection of oncogenic human papillomavirus in sporadic retinoblastoma.* Acta Ophthalmologica Scandinavica, 2003. **81**(4): p. 396-398. DOI: 10.1034/j.1600-0420.2003.00112.x.	Not geographically relevant
112	Paquette, C., et al., *Evidence That Alpha-9 Human Papillomavirus Infections are a Major Etiologic Factor for Oropharyngeal Carcinoma in Black South Africans.* Head and Neck Pathology, 2013. **7**(4): p. 361-372. DOI: 10.1007/s12105-013-0453-0.	HPV data not separated based on gender
113	Passmore, J.A.S., et al., *Papanicolaou smears and cervical inflammatory cytokine responses.* Journal of Inflammation, 2007. **4**. DOI: 10.1186/1476-9255-4-8.	No HPV prevalence data reported
114	Pegoraro, R.J., et al., *P53 codon 72 polymorphism and human papillomavirus type in relation to cervical cancer in South African women.* INTERNATIONAL JOURNAL OF GYNECOLOGICAL CANCER, 2002. **12**(4): p. 383-388. DOI: 10.1046/j.1525-1438.2002.01109.x.	HPV data not separated based on gender
115	Pöllänen, R., S. Vuopala, and V.P. Lehto, *Detection of human papillomavirus infection by non-isotopic in situ hybridisation in condylomatous and CIN lesions.* Journal of Clinical Pathology, 1993. **46**(10): p. 936-939. DOI: 10.1136/jcp.46.10.936.	Location not specified
116	Ramesar, J.E., E.P. Rybicki, and A.L. Williamson, *Sequence variation in the L1 gene of human papillomavirus type 16 from Africa.* Archives of Virology, 1995. **140**(10): p. 1863-1870. DOI: 10.1007/BF01384349.	Non-original research publications
117	Ramesar, J.E., C.M.C. Dehaeck, and A.L. Williamson, *Two different types of human papillomavirus in a patient with carcinomas of the vulva and cervix.* Journal	Location not specified

	of Obstetrics and Gynaecology, 1997. **17**(1): p. 98-99. DOI: 10.1080/01443619750114301.	
118	Richter, K. and G. Dreyer, *Paradigm shift needed for cervical cancer: HPV infection is the real epidemic.* South African Medical Journal, 2013. **103**(5): p. 290-292. DOI: 10.7196/SAMJ.6936.	Non-original research publications
119	Sánchez-Siles, M., et al., *Prevalence of human papillomavirus in the saliva of sexually active women with cervical intraepithelial neoplasias.* Medicina Oral Patologia Oral y Cirugia Bucal, 2020. **25**(2): p. e195-e204. DOI: 10.4317/medoral.23300.	Not geographically relevant
120	Rice, P.S., et al., *High prevalence of human papillomavirus type 16 infection among children.* Journal of Medical Virology, 2000. **61**(1): p. 70-75. DOI: 10.1002/(SICI)1096-9071(200005)61:1<70::AID-JMV11>3.0.CO;2-Y.	Not geographically relevant
121	Schäfer, G., et al., *The role of inflammation in HPV infection of the Oesophagus.* BMC Cancer, 2013. **13**. DOI: 10.1186/1471-2407-13-185.	HPV data not separated based on gender
122	Shapiro, S., et al., *Risk of invasive cancer of the cervix in relation to the use of injectable progestogen contraceptives and combined estrogen/progestogen oral contraceptives (South Africa).* CANCER CAUSES & CONTROL, 2003. **14**(5): p. 485-495. DOI: 10.1023/A:1024910808307.	No HPV prevalence data reported
123	Schmeink, C.E., et al., *Prospective follow-up of 2,065 young unscreened women to study human papillomavirus incidence and clearance.* International Journal of Cancer, 2013. **133**(1): p. 172-181. DOI: 10.1002/ijc.27986.	Not geographically relevant
124	Segondy, M., et al., *Diagnostic value of human papillomavirus (HPV) 16 and HPV18 viral loads for the detection of high-grade cervical intraepithelial neoplasia (CIN2+) in a cohort of African women living with HIV.* Journal of Clinical Virology, 2018. **99-100**: p. 79-83. DOI: 10.1016/j.jcv.2018.01.006.	HPV data not separated based on country
125	Sekee, T.R., et al., *Human papillomavirus in head and neck squamous cell carcinomas in a South African cohort.* Papillomavirus Research, 2018. **6**: p. 58-62. DOI: 10.1016/j.pvr.2018.10.006.	HPV data not separated based on gender
126	Silveira, F.A., et al., *The association of HPV genotype with the regression, persistence or progression of low-grade squamous intraepithelial lesions.* Experimental and Molecular Pathology, 2015. **99**(3): p. 702-706. DOI: 10.1016/j.yexmp.2015.11.001.	Not geographically relevant
127	Singini, M.G., et al., *HPV types 16/18 L1 E6 and E7 proteins seropositivity and cervical cancer risk in HIV-positive and HIV-negative black South African women.*	No HPV prevalence data reported

	INFECTIOUS AGENTS AND CANCER, 2022. **17**(1). DOI: 10.1186/s13027-022-00418-2.	
128	Singini, M.G., et al., *Antibodies against high-risk human papillomavirus proteins as markers for noncervical HPV-related cancers in a Black South African population, according to HIV status.* INTERNATIONAL JOURNAL OF CANCER, 2024. **155**(2): p. 251-260. DOI: 10.1002/ijc.34919.	No HPV prevalence data reported
129	Smits, H.L., et al., *Absence of human papillomavirus DNA from esophageal carcinoma as determined by multiple broad spectrum polymerase chain reactions.* Journal of Medical Virology, 1995. **46**(3): p. 213-215. DOI: 10.1002/jmv.1890460308.	Location not specified
130	Song, S.H., et al., *Interferon-γ (IFN-γ): A possible prognostic marker for clearance of high-risk human papillomavirus (HPV).* Gynecologic Oncology, 2008. **108**(3): p. 543-548. DOI: 10.1016/j.ygyno.2007.11.006.	Not geographically relevant
131	Sudenga, S.L., et al., *Cervical HPV natural history among young Western Cape, South African women: The randomized control EVRI Trial.* Journal of Infection, 2016. **72**(1): p. 60-69. DOI: 10.1016/j.jinf.2015.10.001.	No HPV prevalence data reported
132	Sudenga, S.L., et al., *HPV serostatus pre- and post-vaccination in a randomized phase II preparedness trial among young Western Cape, South African women: The evri trial.* PAPILLOMAVIRUS RESEARCH, 2017. **3**: p. 50-56. DOI: 10.1016/j.pvr.2017.02.001.	No HPV prevalence data reported
134	Stewart Massad, L., et al., *Association of cervical precancer with human papillomavirus types other than 16 among HIV co-infected women.* American Journal of Obstetrics and Gynecology, 2016. **214**(3): p. 354.e1-354.e6. DOI: 10.1016/j.ajog.2015.09.086.	Not geographically relevant
135	Strickler, H.D., et al., *Natural history and possible reactivation of human papillomavirus in human immunodeficiency virus-positive women.* Journal of the National Cancer Institute, 2005. **97**(8): p. 577-586. DOI: 10.1093/jnci/dji073.	Not geographically relevant
136	Swanepoel, P.J., et al., *Cervical squamous intraepithelial lesions and associated cervical infections in an HIV-positive population in Rural Mpumalanga, South Africa.* CYTOPATHOLOGY, 2013. **24**(4): p. 264-271. DOI: 10.1111/j.1365-2303.2012.00998.x.	No HPV prevalence data reported
137	Sworn, M.J., et al., *Squamous intraepithelial neoplasia in an ovarian cyst, cervical intraepithelial neoplasia, and human papillomavirus.* Human Pathology, 1995. **26**(3): p. 344-347. DOI: 10.1016/0046-8177(95)90069-1.	Not geographically relevant

138	Syrjänen, S.M., et al., *In situ DNA hybridization analysis of human papillomavirus (HPV) sequences in benign oral mucosal lesions.* Archives of Dermatological Research, 1987. **279**(8): p. 543-549. DOI: 10.1007/BF00413287.	Location not specified
139	Tanser, F., et al., *Human Papillomavirus Seropositivity and Subsequent Risk of HIV Acquisition in Rural South African Women.* SEXUALLY TRANSMITTED DISEASES, 2013. **40**(7): p. 601-606.	No HPV prevalence data reported
140	Tardío, J.C., et al., *High-risk human papillomavirus determination in formalin-fixed, paraffin-embedded cervical tissue using the roche cobas 4800 system: A comparative study with liquid-based cytology.* International Journal of Gynecological Pathology, 2019. **38**(1): p. 11-16. DOI: 10.1097/PGP.0000000000000467.	Not geographically relevant
141	Tatár, T.Z., et al., *Prevalence of human papillomaviruses in the healthy oral mucosa of women with high-grade squamous intra-epithelial lesion and of their partners as compared to healthy controls.* Journal of Oral Pathology and Medicine, 2015. **44**(9): p. 722-727. DOI: 10.1111/jop.12302.	Not geographically relevant
142	Terada, N., et al., *Human papillomavirus testing and cytology using physician-collected uterine cervical samples vs. self-collected vaginal samples and urine samples.* International Journal of Clinical Oncology, 2022. **27**(11): p. 1742-1749. DOI: 10.1007/s10147-022-02238-1.	Not geographically relevant
143	Tornesello, M.L., et al., *Detection of mucosal and cutaneous human papillomaviruses in oesophagitis, squamous cell carcinoma and adenocarcinoma of the oesophagus.* Journal of Clinical Virology, 2009. **45**(1): p. 28-33. DOI: 10.1016/j.jcv.2009.02.004.	Not geographically relevant
144	Tu, J.J., et al., Molecular variants of human papillomavirus type 16 and risk for cervical neoplasia in South Africa. INTERNATIONAL JOURNAL OF GYNECOLOGICAL CANCER, 2006. 16(2): p. 736-742. DOI: 10.1111/j.1525-1438.2006.00401.x.	No HPV prevalence data reported
145	Van Bogaert, L.J., *Cervical cancer prevention in resource-limited settings with special emphasis on areas of high cervical cancer and human immunodeficiency virus endemicity*, in *Handbook on Human Papillomavirus: Prevalence, Detection and Management*. 2013, Nova Science Publishers, Inc. p. 405-420. ISBN: 978-1-62618-673-6 (eBook).	Non-original research publications
146	van Heerden, W.F.P. and A.W. van Zyl, *Oropharyngeal carcinoma: A sexually transmitted disease.* South African Family Practice, 2010. **52**(6): p. 576-578. DOI: 10.1080/20786204.2010.10874050.	Reported on HPV in men

147	Van Keer, S., et al., *Human papillomavirus genotype and viral load agreement between paired first-void urine and clinician-collected cervical samples.* European Journal of Clinical Microbiology and Infectious Diseases, 2018. **37**(5): p. 859-869. DOI: 10.1007/s10096-017-3179-1.	Not geographically relevant
148	Van De Wijgert, J., et al., Two methods of self-sampling compared to clinician sampling to detect reproductive tract infections in Gugulethu, South Africa. SEXUALLY TRANSMITTED DISEASES, 2006. 33(8): p. 516-523. DOI: 10.1097/01.olq.0000204671.62529.1f.	No HPV prevalence data reported
149	Van Rensburg, E.J., et al., Detection of human papillomavirus DNA with in situ hybridisation in oval squamous carcinoma in a rural black population. South African Medical Journal, 1995. 85(9): p. 894-896. PMCID: 8545753.	HPV data not separated based on gender
150	Visalli, G., et al., *Prevalence of human papillomavirus in saliva of women with HPV genital lesions.* Infectious Agents and Cancer, 2016. **11**(1). DOI: 10.1186/s13027-016-0096-3.	Not geographically relevant
151	Wang, J.S., et al., *Human papillomavirus in cyclophosphamide and diverticulum-associated squamous cell carcinoma of urinary bladder: A case report.* Chinese Medical Journal (Taipei), 1996. **57**(4): p. 305-309. PMCID: 8705886.	Not geographically relevant
152	Wang, Y., et al., *Non-Lactobacillus-Dominant and Polymicrobial Vaginal Microbiomes Are More Common in Younger South African Women and Predictive of Increased Risk of Human Immunodeficiency Virus Acquisition.* Clin Infect Dis, 2023. **76**(8): p. 1372-1381. DOI: 10.1093/cid/ciac938.	Not geographically relevant
153	Wei, Z.T., et al., *Depiction of Vaginal Microbiota in Women With High-Risk Human Papillomavirus Infection.* Frontiers in Public Health, 2021. **8**. DOI: 10.3389/fpubh.2020.587298.	Not geographically relevant
154	Weinberger, P.M., et al., *Human papillomavirus-active head and neck cancer and ethnic health disparities.* Laryngoscope, 2010. **120**(8): p. 1531-1537. DOI: 10.1002/lary.20984.	Not geographically relevant
156	Wensveen, C., et al., *Detection of cervical intraepithelial neoplasia in women with atypical squamous or glandular cells of undetermined significance cytology: A prospective study.* Acta Obstetricia et Gynecologica Scandinavica, 2003. **82**(9): p. 883-889. DOI: 10.1034/j.1600-0412.2003.00231.x.	Not geographically relevant
157	Williamson, A.L. and E.P. Rybicki, *Detection of genital human papillomaviruses by polymerase chain reaction amplification with degenerate nested primers.* Journal of Medical Virology, 1991. **33**(3): p. 165-171. DOI: 10.1002/jmv.1890330305.	Location not specified

158	Woelber, L., et al., *Oral Human Papillomavirus in Women with High-Grade Cervical Intraepithelial Neoplasia.* Journal of Lower Genital Tract Disease, 2017. **21**(3): p. 177-183. DOI: 10.1097/LGT.0000000000000313.	Not geographically relevant
159	Woods, M., et al., *Detecting human papillomavirus in ocular surface diseases.* Investigative Ophthalmology and Visual Science, 2013. **54**(13): p. 8069-8078. DOI: 10.1167/iovs.13-13140.	Not geographically relevant
